# How does technological system design affect value creation? A systematic literature review of digital co-production

**DOI:** 10.1007/s43508-022-00051-0

**Published:** 2022-10-28

**Authors:** Rui Mu, Yuting Wang, Haoqi Song

**Affiliations:** 1 Faculty of Humanities and Social Sciences, Dalian University of Technology, Dalian, 116024 China; 2 JCU Singapore Business School, James Cook University, 149 Sims Drive, Singapore, 387380 Singapore

**Keywords:** Co-production, Value creation, Digital technology, Design factors

## Abstract

The existing studies on co-production display two research gaps. First, most studies focus on non-digital/offline co-production and value creation; little attention has been paid to value creation of digital/online co-production cases. Second, traditional co-production studies examine political, organizational, administrative, and personal factors that influence co-production. However, few studies investigate how technological factors will affect co-production in terms of value creation. To bridge the gaps, this article conducts a systematic literature review of 52 articles. The review results distill seven technological factors and five value categories from digital co-production cases. It further examines how these technological factors affect the creation of various value categories. Based on the review results, this article proposes a future research agenda on digital co-production.

**Supplementary Information:**

The online version contains supplementary material available at 10.1007/s43508-022-00051-0.

## Introduction

Co-production is an umbrella concept which captures the extensive collaborative activities between governments^1^ and citizens^2^ that occur in the production cycle of public services (Alford, 2014; Bovaird, 2007). Traditionally, co-production takes offline forms. Nowadays, the digital transformation, driven by the emergence of information communication technologies and the development of the internet, has enabled governments to design online opportunities for citizens to engage with and contribute resources (e.g., knowledge, ideas, time, energy) to the production of public services, i.e., the so-called digital co-production (Mu & Wang, 2022; Loeffler, 2021: 135).

The extant studies on co-production largely concentrate on non-digital forms and pay substantial attention to exploring the political, organizational, administrative, and personal factors that influence co-production (e.g., Brandsen et al., 2018; Loeffler, 2021; Pestoff, 2019). For instance, the literature review undertaken by Voorberg et al. (2015) shows that on the governments’ side, the lack of communication infrastructure with citizens, the passive attitude of politicians, the risk-averse and conservative administrative cultures influence co-production, and on the citizens’ side, the lack of civic obligation, inadequate social capital, and the lack of self-efficacy hinder co-production.

The existing studies on co-production display two important research gaps. First, little attention has been paid to digital/online forms of co-production and very few analyses focus on the technological factors that affect co-production. Only a few studies have mentioned the macro-level impacts of digital technologies on co-production. For example, Lember et al. (2019) analyzed the positive and negative effects of sensing, communication, processing, and actuation on citizens’ motivation to engage with public services but did not mention how these technologies should be designed into user-friendly systems. Similarly, Kattel et al. (2020) examined the conditions under which digital technologies enhance or hinder the interactions between governments and citizens but did not clearly explain how the interactions could be structured through technological system design. To bridge this gap, our systematic review focuses on digital co-production programs and identifies the design factors of the digital technological systems. Therefore, the first research question (RQ1) reads as:

*RQ1: What are the technological system design factors that influence digital co-production?*

Besides, the second limitation of the extant studies is that few efforts have been devoted to exploring the relationship between technological system design and value creation. In other words, the question of how digital co-production’s technological system design will influence value creation still remains unaddressed. Osborne et al. (2021) argue for the need to go beyond appreciating co-production as a stand-along process and promote a value creation view to examine the co-production outcome. Nevertheless, the link between co-production’s technological system design factors and value creation is still missing. Therefore, to fill this gap, this present article aims to answer the second research question (RQ2):

*RQ2: What value categories can digital co-production create, and how will the technological system design factors influence value creation?*

To answer the research questions, we conducted a systematic literature review (SLR) of articles focusing on digital co-production by using the PRISMA approach (Preferred Reporting Items for Systematic Reviews and Meta-Analyses). This SLR has both theoretical and practical significance. Theoretically, our review provides new insights on digital co-production in terms of the technological system design factors and their links with value creation. In practice, our review of the technological system design factors provides a timely reminder for public managers, administrators, and professionals to design a digital architecture/interface that is easier to use, attractive for citizens, and ultimately beneficial for value creation.

This article is organized as follows: Sect. 2 presents the methodology of this review. Section 3 offers an initial general description of our review results, and Sect. 4 reports the specific review results on the technological design factors, the value categories of digital co-production cases, and the causal links between the technological factors and value creation. Section 5 summarizes the review findings, proposes a future research agenda on digital co-production, and points out the limitations of this review.

## Methodology

In this SLR, we used the standardized way of PRISMA (Moher et al., 2010) to identify relevant publications on digital co-production and use these publications as bases to examine what technological system design factors are at play in digital co-production programs, what value categories are created, and how these factors influence value creation of the digital co-production programs. The PRISMA approach consists of several explicit steps of setting eligibility criteria, formulating search strategies, selecting records, and clarifying coding strategies. We will outline how we operationalized these steps in greater details below.

### Eligibility criteria

The identification of relevant publications is based on six eligibility criteria:

*(1) Field* The research topic must concentrate on collaboration between governments and citizens. Some collaboration between governments and businesses, for instance, is not considered. However, we extend the “governments” to the organizations and professionals mandated by or acting on behalf of governments; and we use “citizens” as a representative group for service users and communities.

*(2) Topic* Articles to be included in the review need to focus on digital co-production, so non-digital co-production cases are not considered. However, it is not meaning that we only focus on those articles with “digital co-production” in their titles or abstracts. We examine whether the collaborative activities occur between governments and citizens, whether the goals of the collaborations are related to public services, and whether the collaborations take place via digital technologies.

*(3) Study design* Only articles with empirical digital co-production programs were included in our review. Conceptual work was not included. This is because our research purpose is to extract the design factors from the technological systems of digital co-production programs in empirical cases. Therefore, the academic work discussing digital co-production at the conceptual level cannot provide us with empirical evidence and thus cannot serve for our research purpose.

*(4) Language* Given the practical difficulties of translation, only articles written in English were included in the review.

*(5) Publication year* Articles published in all past years are searched and included, that is, prior to June 30, 2021.

*(6) Publication type* Only international peer-reviewed journal articles from the ISI Web of Science Core Collection database were included in this review.

### Search strategies

We established five strategies to search relevant articles:

(1) *Formulating keywords for initial article searches.*

The foremost difficulty of this SLR is to identify what collaboration activities between governments and citizens belong to digital co-production. To overcome this difficulty, we applied Loeffler’s (2021) “Four Co’s” model as a starting point to create relevant “keywords” for initial article searches. Loeffler’s model consists of four types of digital co-production: digital co-commissioning, digital co-design, digital co-delivery, and digital co-assessment (Table 1).

**Table 1:** Digital modes of co-production (Adapted from Loeffler, 2021)

Digital co-production modes	Brief description	Digital technologies adopted	Examples	Keywords for article searches
Digital co-commissioning	Citizen involvement to identify and prioritize proposals	Digital platform Online comment system Visualization and GIS	Online urban planning Online participatory budgeting	“urban planning” OR “participatory budgeting”
Digital co-design	Citizen involvement to input innovative ideas and offer novel solutions	Digital platform Online data sources	Crowdsourcing Hackathon	“crowd$funding” OR “crowdsourcing” OR “hackathon*
Digital co-delivery	Citizen involvement to implement public programs, manage public facilities, form peer support networks	Digital platform Social media	Digital volunteer networks in emergency and disaster management Online peer support E-health	“emergency OR crisis OR disaster” OR “e-health”
Digital co-assessment	Citizen involvement to evaluate public services and report problems	Web-based technology (e.g., government portal) Mobile Apps	Online citizen reporting Remote health monitoring	“citizen reporting” OR “remote health monitoring”

Digital co-commissioning refers to governments working with citizens through digital technologies to identify and prioritize public service outcomes. Here, citizens act as strategic thinkers and make voices to shape public service agenda. Within this mode, Loeffler identified two typical examples, online urban planning (i.e., governments provide citizens with the visualized 3D scenarios of new neighborhood developments, and citizens can make comments or prioritize different development proposals) and online participatory budgeting (i.e., governments construct a digital platform to allow citizens vote on proposals about government spending and thus commission public budget). Therefore, we created “urban planning” and “participatory budgeting” as two keywords for initial article searches.

Digital co-design refers to citizens providing their creative and novel ideas for governments through online systems, in order to improve the pathways leading to service outcomes. Within this mode, Loeffler identified another two typical examples, crowdsourcing (i.e., governments use online platforms to crowdsource new ideas from citizens) and hackathons (i.e., governments organize competitive events and offer data to software design companies or individuals to creative new APPs for specific public service). This provided us with new keywords for initial article searches, including “crowd$funding,” “crowdsourcing” and “hackathon*”.

Digital co-delivery involves a range of citizen actions, such as the co-implementation of public programs, co-management of public facilities, co-performing peer support, and taking joint action to improve public services and outcomes. In this mode, governments view citizens as asset-holders and want to lever in their capabilities, skills, time, and resources to improve public services or to solve public problems. Loeffler mentioned that emergency management and e-health are two typical cases in this field. In emergency management, governments use social media to coordinate citizens’ collective action for disaster response. Or, governments build up online platforms to match people who are at risk and volunteers who can help. In e-health, citizens use the Internet and other electronic media to access health services, and to communicate with population members, health professionals, health insurers, and policy makers. This brought us with some new keywords for initial article searches, including “emergency OR crisis OR disaster” and “e-health.”

Digital co-assessment refers to citizens using online channels to express their satisfactions on public services, report problems, and evaluate service performance. In this mode, governments invite citizens to make voices on service quality because citizens often know better than professionals whether a service performs good or presents any problems. Loeffler identified two relevant cases in this mode, online citizen reporting and online service monitoring and evaluation, which enables us to formulate two new keywords for initial article searches: “citizen reporting” and “remote health monitoring.”

(2) *Ensuring that the articles talk about citizen participation.*

Not all digital co-production articles found by the above-mentioned keywords involve discussions on citizen participation. For example, some articles on e-health focus on secure and privacy-preserving data sharing in e-health systems and do not touch upon any discussions on how governments and citizens co-produce health services. The same problem also happened to some articles on urban planning and disaster response. Therefore, our second strategy is to ensure that the found articles include discussions on citizen participation. To do so, we used the set of terms “citizen engag* OR citizen participat* OR public engag* OR public participat* OR co$produc* OR co$creat* OR open innovation OR collaborative innovation OR open government OR we-govern*” to narrow down the initial identified articles.

(3) *Ensuring that the articles talk about technological systems of digital co-production programs.*

Although the articles found are about digital co-production, not all of them introduce or explain how the technological systems of the digital co-production programs look like and work, thus not fitting for our research purpose. In order to exclude those inappropriate articles, we subsequently searched within the newly obtained results to identify articles that touch upon the discussions on the technological systems by using the terms “e-govern* OR e-collaboration OR e-participat* OR digital OR online OR social media OR ICT OR information communication technolog* OR internet OR web.”

(4) *Ensuring that the articles fit our research purpose by screening article titles and abstracts.*

In this step, we screened the article titles and abstracts to further assess the relevance of the topics of the records and to ensure that the distilled articles fit our research purpose.

(5) *Ensuring that the articles fit our research purpose by reading the full texts.*

Finally, we read the full texts to assess the relevance of content in records. At this stage, we excluded the articles that do not discuss any system design factors for digital co-production or that do not involve any discussions on value creation.

### Record selection

**Fig. 1:**
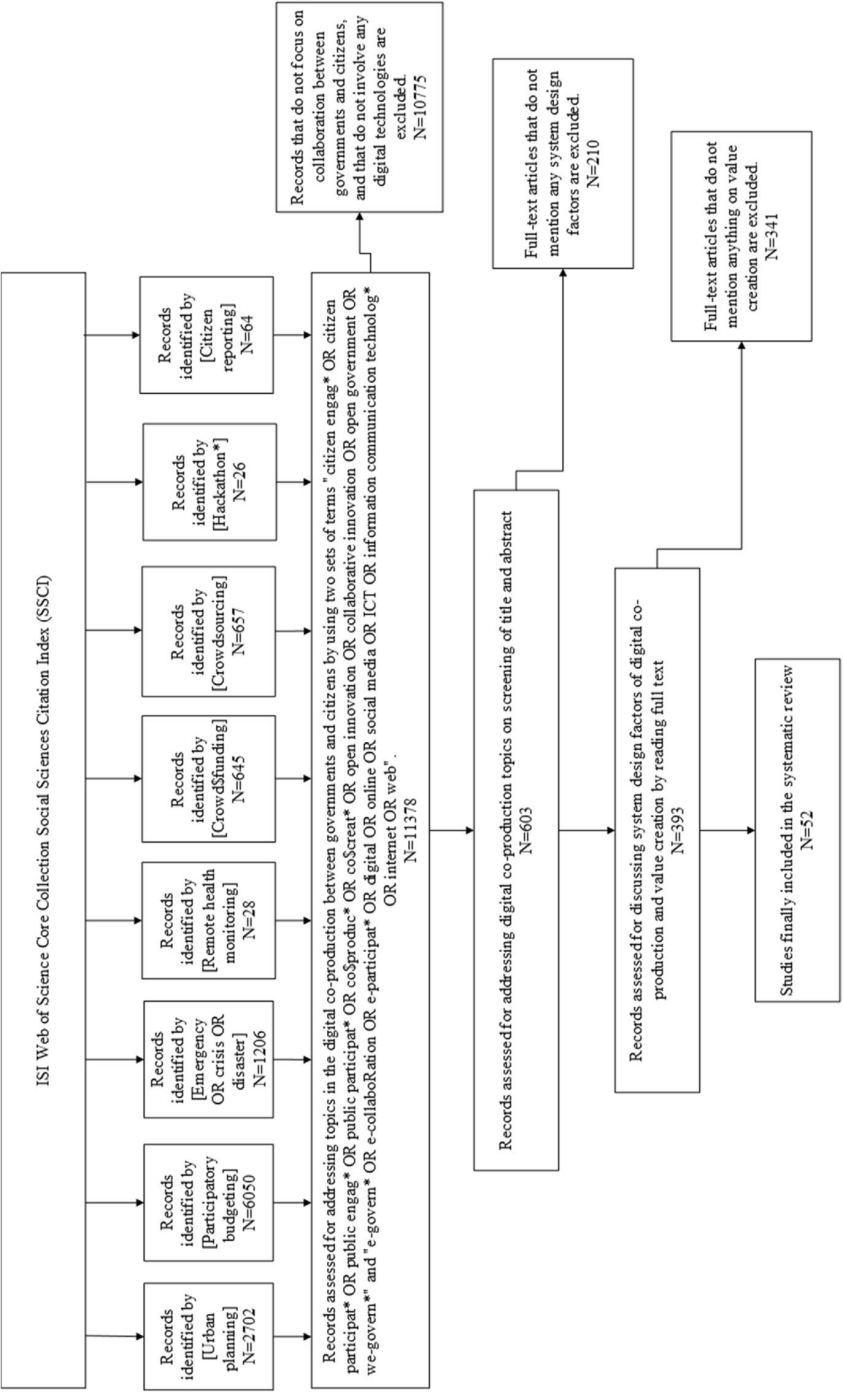
The PRISMA flow diagram

Following the eligibility criteria and the search strategies, 52 studies were finally included in our systematic review. Figure 1 illustrates our assessment and selection process. Appendix 1 presents the basic article information, including authors, publication years, publication journals, countries under analysis and government layers, and digital technologies adopted in the empirical cases.

### Coding strategies

IN this SLR, we adopted a three-level coding procedure. The first level is information extraction. We extracted textual data (e.g., words and sentences) describing the technological systems’ characteristics, appearance, functions, rules, etc. by reading the full texts of the sample articles. In order to examine how the technological system design factors influence value creation, we also paid attention to the statements and opinions in the sample articles on the benefits, achievements, advantages, performance, satisfaction, and outcomes of the digital co-production programs. The second level is open coding. During this process, we analyzed the extracted textual data and broke them into discrete parts; then we continuously compared and contrasted similar discrete parts and created “codes” to label them. The third level coding is axial coding. In this process, we read over our codes developed in open coding, drew connections between the codes, and grouped similar codes into new categories. A category could be created based on an existing code, or a new more abstract category can be developed that encompasses several different codes. The results of the whole coding process are shown in Appendix 2 of this article.

Three researchers conducted the coding process. In each coding procedure, the researchers first worked individually and then came together at the end of each procedure. The extracted textual data, open codes, and axial codes from the three researchers were cross-checked and synthesized into commonly agreed datasets. When disagreements happened, the three researchers had discussions until consensus was reached.

## General description

### Publication year

**Fig. 2:**
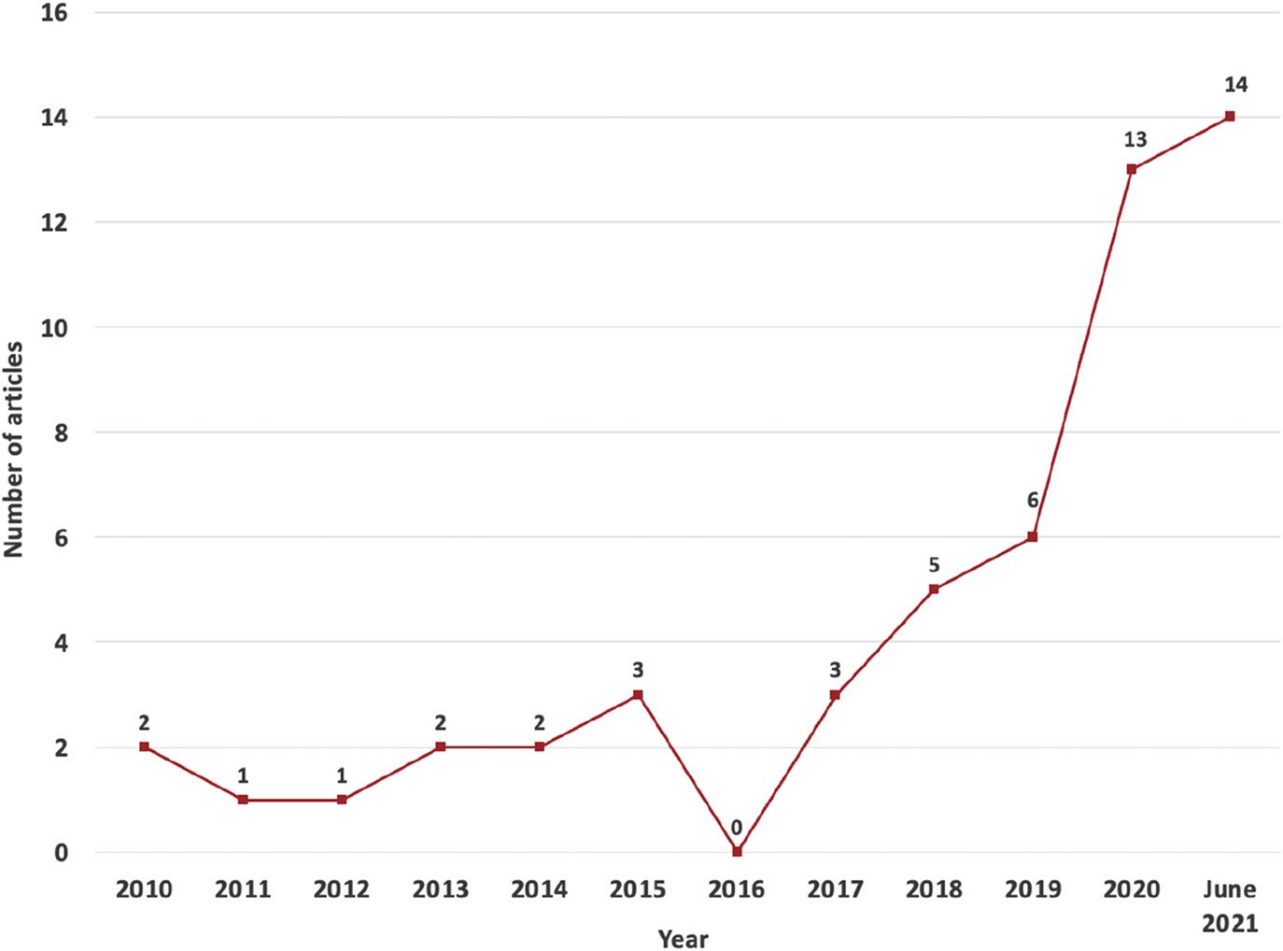
Number of articles and publication year

Figure 2 shows the evolution of the number of articles published on digital co-production until June 2021. Although articles published in all past years are searched, the earliest articles included in our sample appeared in 2010. Both articles are about digital co-production in urban planning. One assesses public participation in a digital urban planning program in a Brazilian city, and another focused on the interactive tools designed for improving public participation in an online visualized urban planning program. From 2010 to 2017, the number of articles remained fairly constant, at roughly 1.75 articles per year (14 articles in 8 years). However, since 2018 it has witnessed a remarkable increase, with 11 articles published annually (38 articles in 3.5 years). This trend indicates that digital co-production for public services has become an increasingly popular research topic among scholars.

### Journals and categories

**Fig. 3:**
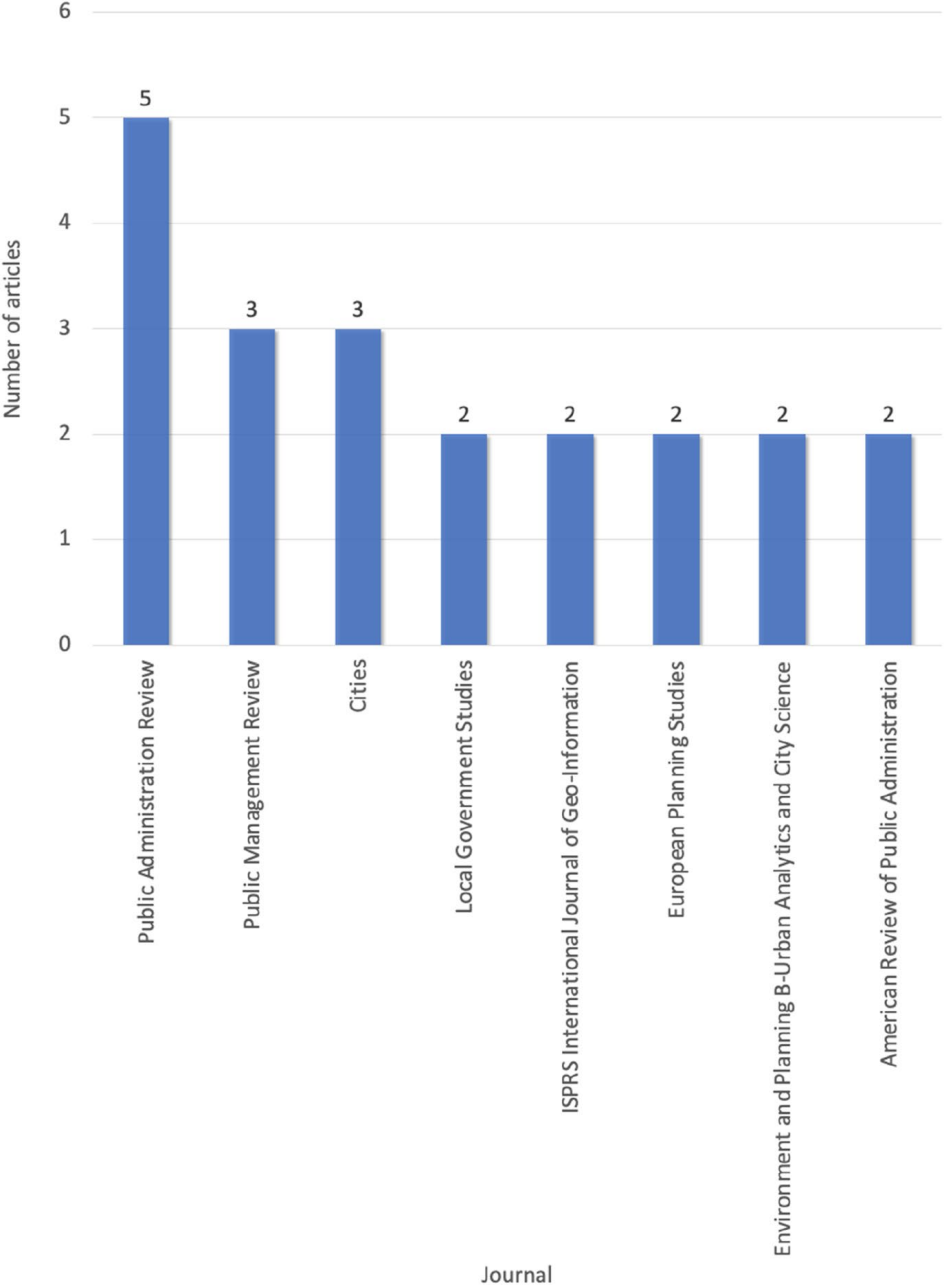
Publication journals

**Fig. 4:**
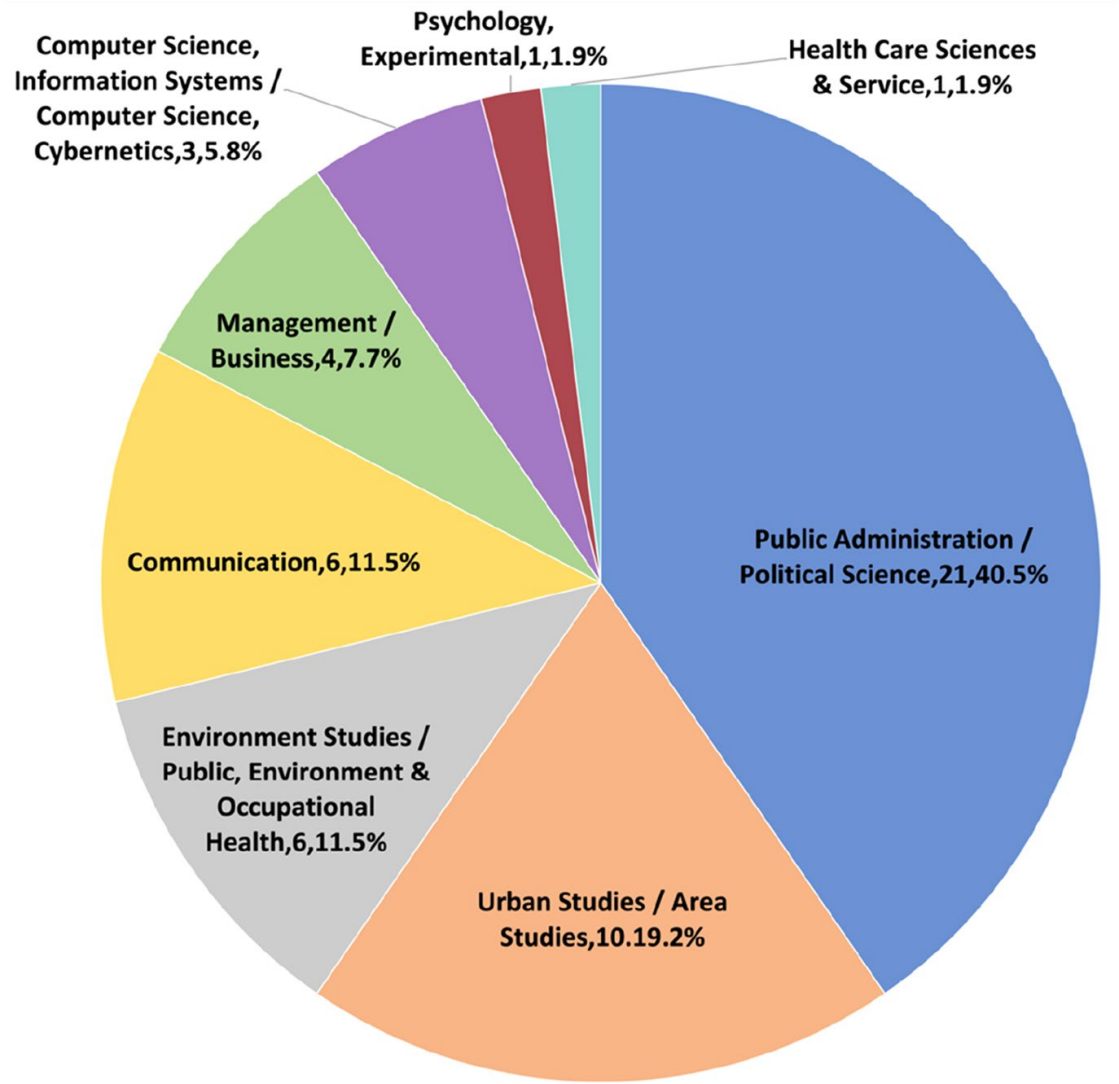
Categories of journals

The reviewed articles are published in 39 different journals; only those (*n* = 8, 20.5%) publishing at least two digital co-production articles are presented in Fig. 3. The journals are covered by a variety of categories (Fig. 4). As can be seen, 40% of the journals are in the Public Administration and Political Science categories, and 60% touch upon the categories of Urban Studies and Area Studies, Environmental Studies, Communication, Business and Management, Computer Science and Information Systems, etc. The diverse distribution of journals and categories implies that digital co-production is a multidisciplinary topic.

### Countries under analysis and government layers

**Fig. 5:**
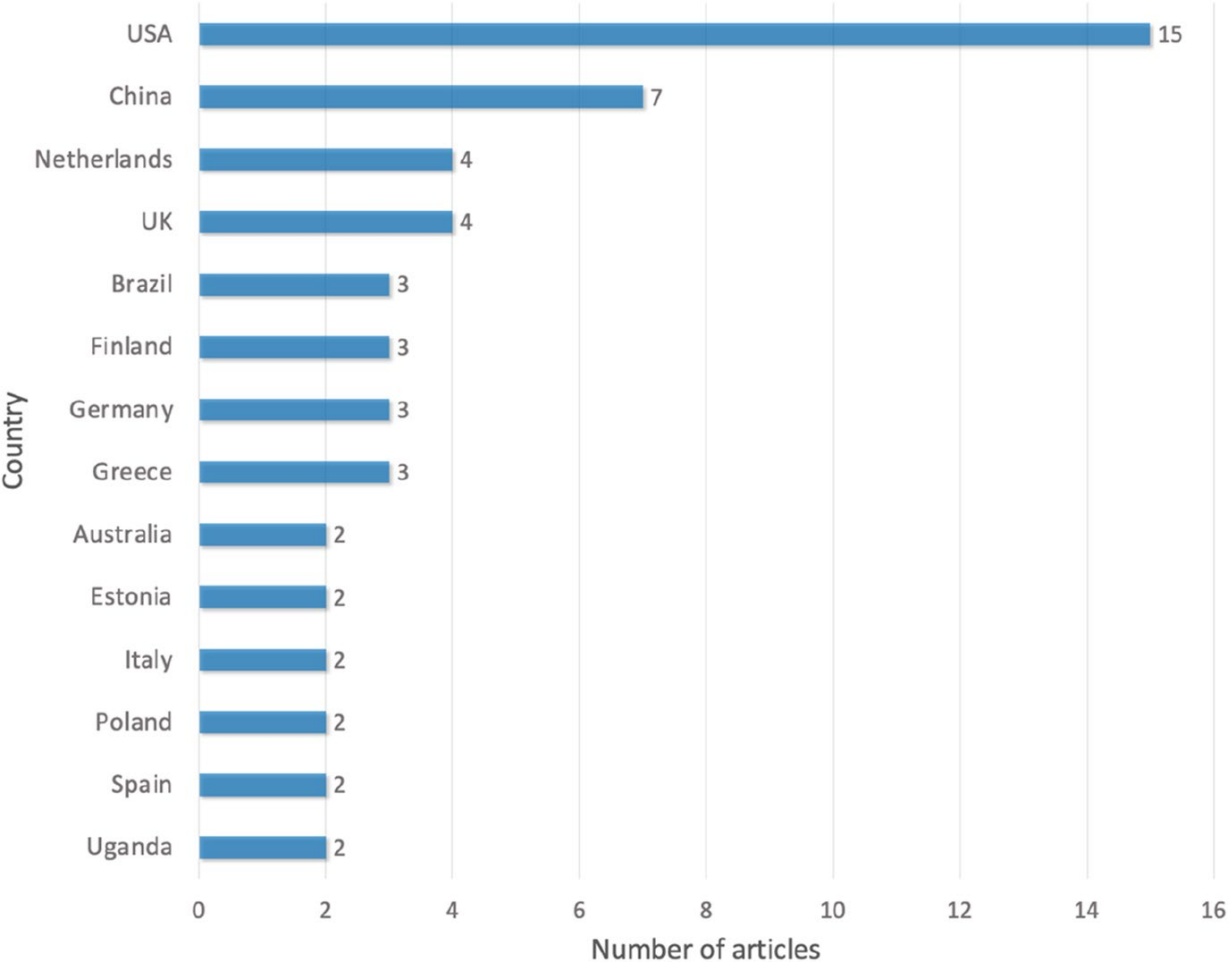
Countries under analysis

The digital co-production cases in the sample articles touch upon 23 countries; the countries that appear at least twice are presented in Fig. 5. The U.S. is the most frequent country to be studied (15 articles study the U.S. cases). China is in the second position (7 articles), followed by the Netherlands and the UK (4 articles, respectively). In addition, the largest group of digital co-production articles were conducted on the local government level (*n* = 42, 80.8%), followed by the central government level (*n* = 9, 17.3%) and the international level (i.e., the European Union) (*n* = 1, 1.9%).

### Digital technologies adopted

**Fig. 6:**
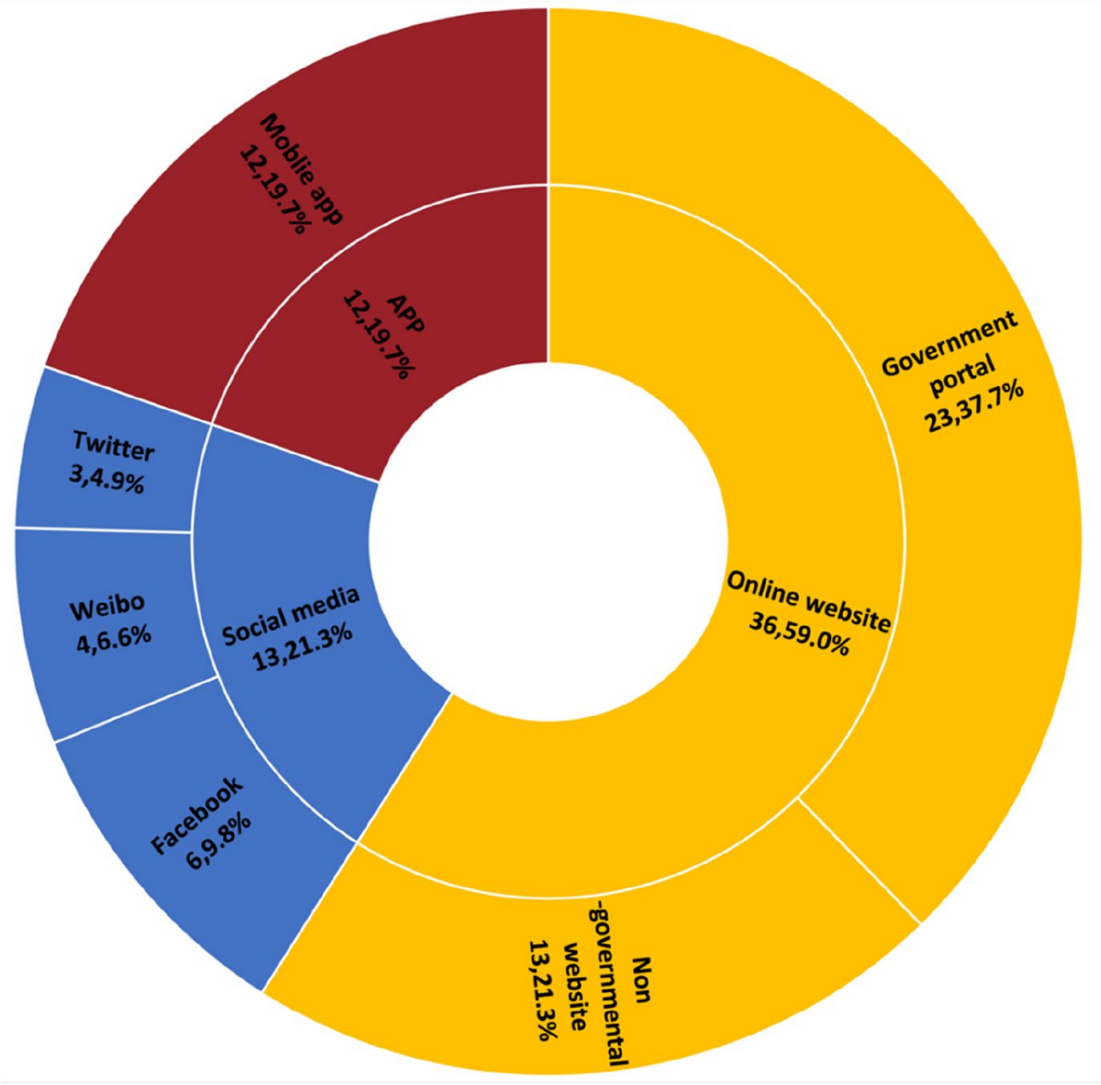
Digital technologies adopted

Digital co-production for public services mainly depends on the use of three technologies in our reviewed articles: mobile APP, social media, and online website (Fig. 6). The most frequently used technology is online website (*n* = 36, 59.0%), which contains government portals (*n* = 23, 37.7%) and nongovernmental websites (*n* = 13, 21.3%). The second type of technology used is social media (*n* = 13, 21.3%), including Facebook (*n* = 6, 9.8%), Twitter (*n* = 3, 4.9%) and Weibo (*n* = 4, 6.6%). The third type of technology is various mobile APPs (*n* = 12, 19.7%).

## Review results and analyses

### Technological system design factors of digital co-production

This section presents the technological system design factors of the digital co-production programs in our reviewed articles. We coded them online marketing factors, incentivizing factors, “ease-of-use” factors, government-to-citizen communication factors, citizen-to-citizen interaction factors, online/offline integration factors, and data collaboration factors (see Appendix 2 for the detailed coding process).

#### Online marketing factors

Our reviewed articles indicate that some technological systems of digital co-production programs are designed with marketing endeavors. These endeavors aim at establishing, developing, and maintaining good online relationships between governments and citizens. First, we found that in some crowdfunding cases (e.g., Colasanti et al., 2018) governments conduct *prior online surveys* to find out how much citizens know about the crowdfunding program, citizens’ attitudes toward the program, and their willingness to participate. Second, in the crowdsourcing programs (e.g., Mergel & Desouza, 2013; Royo & Yetano, 2015), governments *advertise* the co-production programs through press releases, social media, government portals, and e-mail to relevant and affected stakeholders, in order to attract public attention, invite notable individuals, and keep constant media mentions and coverage. Third, governments also market co-production programs through holding *online competition events*, in order to attract citizens to put forward solutions, suggestions, and ideas on public problems (e.g., Cinderby et al., 2021; Mergel, 2018).

#### Incentivizing factors

Our reviewed articles suggest that some technological systems involve incentivizing strategies that governments use to motivate citizens to participate in co-production and contribute time, energy, intelligence, expertise, and money. First, some governments put the notice of *monetary rewards* onto the technological interfaces, indicating that citizens will be given cash awards or gift cards, etc. for their participation (e.g., Moon, 2018; Pihlajamaa & Merisalo, 2021). However, scholars indicate that the backers involved in co-production programs may not expect monetary rewards; instead, they regard reputational and emotional rewards as more valuable since they are initially driven by a sense of engagement and belonging to the local territories. Thus, in some local campaign programs (e.g., de Crescenzo et al., 2021), governments embed *reputational (non-monetary) incentives* into the technological systems, which offer the winners acknowledgment of their win in the form of a press release to the media and an announcement on the government portal (also see Brabham, 2012).

#### “Ease-of-use (EoU)” factors

“Ease-of-use (EoU)” factors refer to those technological designs that make the system interfaces easy to use and user-friendly. First, some system interfaces provide *convenient registration and login* procedures for citizens (e.g., Antoniou et al., 2020); after registration and login, citizens can choose their *prefer language (or non-professional language)* and a webpage is automatically created where the citizens can view all relevant information on the co-production programs they participate (e.g., Leston-Bandeira, 2019); moreover, some systems are designed with a *“little helper”* that gives suggestions on how to use the systems (e.g., Poplin, 2014); and some systems also provide *push notifications* to the citizens whenever signs of progress are made on the programs (e.g., Wilson et al., 2019). Second, a considerable number of technological systems in urban planning programs are linked to *Geographical Information System* (GIS) and armed with *visualization* tools. In these cases, citizens are provided with online maps to mark spots and make comments on particular locations, in order to report problems (e.g., potholes in the streets, broken public lights, broken public play equipment, litter on the streets) of public space (Kurniawan & de Vries, 2015).

#### Government-to-citizen communication factors

Our reviewed articles indicate that the technological systems of digital co-production programs all contain communication rules between governments and citizens. First, in emergency and crisis management cases, the technological systems are often embedded with *the functions of “@”* to prompt certain accounts and *“#”* to post hot topics to create dialogic loops between governments and citizens (Young et al., 2018). Second, the technological systems are usually designed with *media richness* features, including the functions of playing videos, inserting pictures, photos, and hyperlinks, and displaying *narratives/stories* when creating a post (e.g., Liu et al., 2020). If the media richness is low, then governments might adopt the *narrative strategy* to communicate with citizens (e.g., Ngai et al., 2020). Third, the technological systems set *government replies* as a mandatory procedure to proceed with citizen messages (e.g., Buntaine et al., 2021; Jankowski et al., 2019), even when the governments reject citizens’ proposals (e.g., Li et al., 2020).

#### Citizen-to-citizen interaction factors

Our reviewed articles also indicate that some technological systems include the interaction rules between citizens themselves. These citizen-to-citizen interaction rules include the *possibilities of scoring, ranking, and commenting* on each other’s ideas and proposals and *giving “likes”* to others’ messages and solutions. For instance, in the cases of crowdsourcing bike station locations, transit routes, marketplaces, and communities, the technological systems open the functions of proposal scoring, commenting, and ranking between citizens (Brabham, 2012; Griffin & Jiao, 2019; Meijer, 2011; Poplin, 2014). Besides, in the field of e-budgeting, Mærøe et al. (2021) showed that the technological platform of Tartu allows citizens to score and vote the proposals on how to spend 1% of the city’s investment budget.

#### Online-offline integration factors

Online-offline integration refers to the strategies that combine online and offline activities in one co-production program. First, the technological system of online co-production allows information feedback from offline interactions and discussions. In an urban planning case, Satorras et al. (2020) showed that in the collection phase of urban design proposals, two offline workshops were held by planning agencies to solicit opinions from citizens, and then the facilitators summarized and published the consensus reached offline in the digital platform, where citizens could still comment and value them. Besides, in some cases, the solutions and proposals citizens brought forward online are taken offline for further elaboration and assessment. This happened in a South Korean case where online policy suggestions from citizens are later reviewed, assessed, and elaborated in offline meetings by government officials (Moon, 2018).

#### Data collaboration factors

Our reviewed articles show that the technological systems of digital co-production programs are usually designed with data collaboration efforts. First, data sharing between governmental departments is a necessary design condition for technological systems. For instance, in the e-residency program in Estonian, multiple government departments built up the data exchange architecture (the so-called X-road) and collaborated for the program in order to allow the foreign residents to help themselves to handle working, living, and taxation affairs (Kattel et al., 2020). Second, the technological system allows NGOs to visit the system and extract data from the system to produce readable and understandable datasets for citizens. For instance, in the case of the data reuse co-production project in Quebec, it is data intermediaries that helped the government to produce standardized and unified datasets, which makes it convenient for citizens to reuse the data and to produce innovations (Boudreau, 2021).

### Value creation of digital co-production

This section presents the value categories created by the digital co-production programs in our reviewed articles. In the axial coding process, we labeled them experiential value, performance value, whole-life value, capacity value, and administrative value (see Appendix 2 for the detailed coding process).

#### Experiential value

The first value category distilled from our reviewed articles touches upon the satisfaction of citizens with their experience of using online public services (e.g., Meijer, 2011; Brabham, 2012; Pieper & Pieper, 2015). As argued by the service dominant logic theory that services have no intrinsic value; only when a service is used, it will produce value for the user (i.e., value-in-use). Our reviewed articles also confirm this point; such value is a kind of short-term experiential value produced in the moment of service consumption. Thus, in this SLR, we use the term “experiential value” to label this value category.

#### Performance value

Our reviewed articles also show that some digital co-production programs can create better performance of public services. For instance, public services become more efficient (doing more with less) after online public participation (e.g., Kattel et al., 2020; Mourafetis & Potsiou, 2020; Paul & Sosale, 2020), and the service solutions are more innovative (e.g., Mergel, 2018; Mergel & Desouza, 2013), and feasible (e.g., Royo & Yetano, 2015). Different from the short-term experiential value, this improved performance of public services can be reached and assessed only after services are delivered. Thus, this value category is a medium-term value and we call it “value-after-use.”

#### Whole-life value

The third value category surfacing out of our reviewed articles entails the influence of public services on the whole-life experience of a service user. For example, as Osborne et al. (2021) argued, high-school education does not simply impart knowledge to children but will determine how the children will subsequently construct their personalities. Therefore, this value category focuses on individual service users and refers to the long-term invasive influence on users’ emotion, psychology, and whole-life experience (e.g., Bugs et al., 2010; Meijer, 2011; Poplin, 2014; Satorras et al., 2020; Wilson et al., 2019). In this SLR, we coded this value category “whole-life value.”

#### Capacity value

The fourth value category distilled from our reviewed articles relates to the value created by public services by enhancing users’ abilities to change or to solve their own needs and problems in the future. Through engaging with public services, the users can accumulate collaboration experience, obtain more knowledge, learn, and get trained, so that they can use the newly obtained skills and knowledge to solve their own problems, not merely depending on the solutions provided by governments (e.g., Liu et al., 2020; Meltzer et al., 2018; Wukich, 2021; Young et al., 2018). In this SLR, we used the term “capacity value” to denote this value category.

#### Administrative value

The fifth value category relates closely to what we know early on “administrative values” (Hood, 1991: 10), which embody the way that governments’ administrative departments behave and the principles they carry out routine tasks and make decisions. Our reviewed articles show that digital co-production programs can generate various administrative values, including engagement (Mergel, 2018), equality (Clark et al., 2013), legitimacy (Moon, 2018), transparency (O’Brien et al., 2017), accountability (Young et al., 2018), and trust (Antoniou et al., 2020). In this SLR, we use the term “administrative value” to code this value category that digital co-production adds to the administration departments and the nation’s governance system as a whole.

### The causal links between the technological system design factors and value creation

**Fig. 7:**
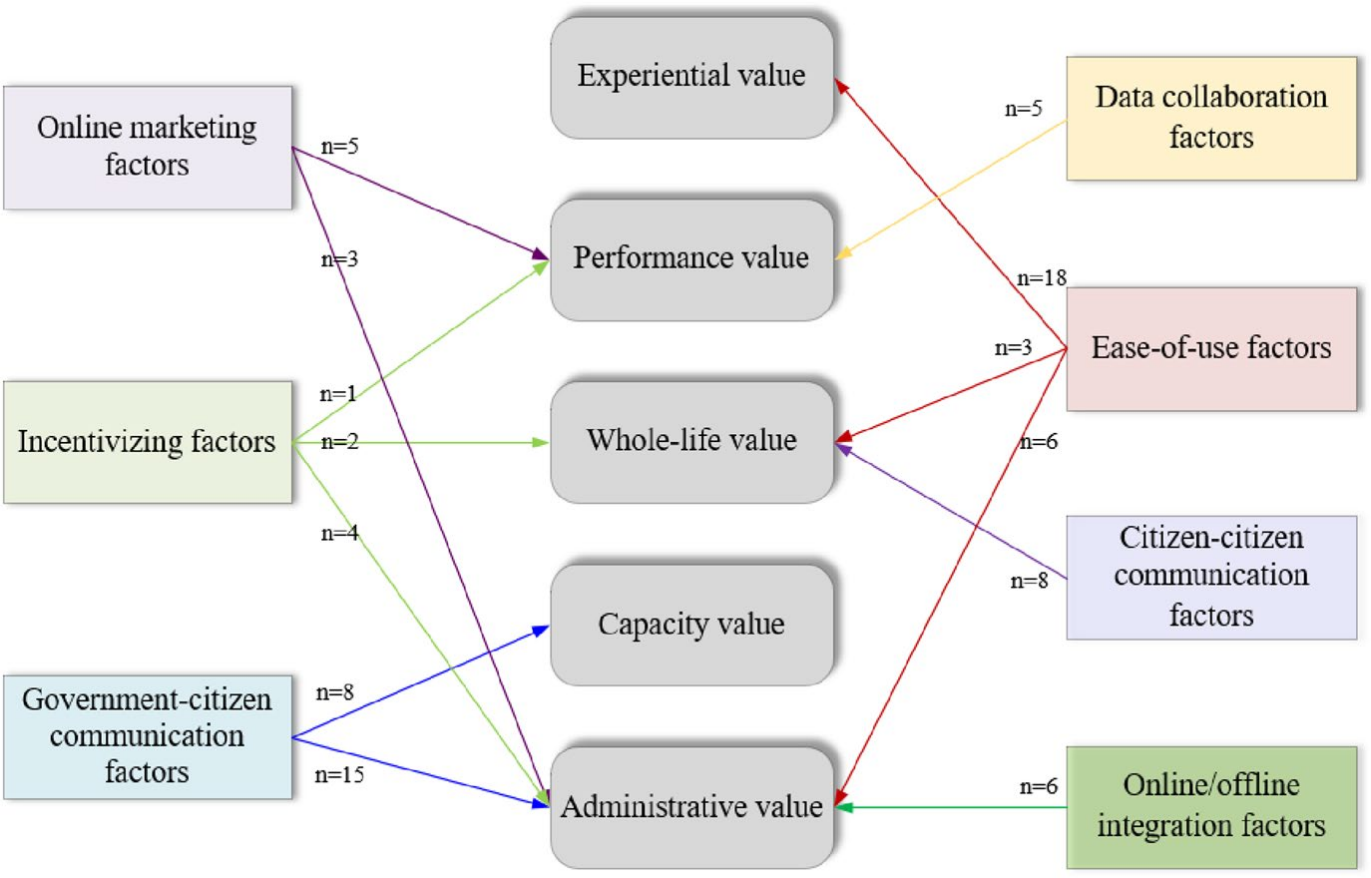
Causal links between system design and value creation

IN the previous two sections, we reported the technological system design factors and the value categories identified from the coding process. In this section, we present the causal links between the design factors and the value categories. We identify the existence of a causal link when a technological system design factor and a value category concurrently appear in one digital co-production program. Figure 7 shows the causal links (with frequency, *n*) between the identified design factors and the value categories.

#### Ease-of-use (EoU) factors bring about experiential value

*Our reviewed articles reveal a causal link between technological interfaces’ EoU factors and citizens’ experiential value creation.* This is because ease-of-use design will reduce the demand for citizens’ efforts to gain new navigational skills, increase citizens’ perception of “ease-of-use” on the digital interfaces, and eventually enhance citizen satisfaction and bring about a good user experience with the system. For instance, in the cases of co-producing urban plans, integrating GIS and visualization into the technological interfaces makes citizens more absorbed in using the system (Bugs et al., 2010; Jankowski et al., 2019; Kahila-Tani et al., 2019; Wang et al., 2021). Citizens virtually glance over the urban environment and operate on the online map as if they were planning in the real physical locations, making the use of the system fun and pleasant (Jankowski et al., 2019; Kahila-Tani et al., 2019; Poplin, 2014; Wu et al., 2010). In addition, in the e-petition and online cadastral management cases, the convenient registration and login procedures, as well as the embedment of a “little helper” in the system interface, may increase citizens’ belief that using the system is easy and learning to navigate the system is free of effort.

#### Online marketing and data collaboration contribute to performance value

*First, our reviewed articles indicate a causal link between governments’ online marketing strategies and the creation of performance value.* This is because, according to the service ecosystem perspective, the creation of performance value in the medium and long-run is dependent on the quality of relationships between the various elements within the ecosystem, including users, providers, technologies, and even contextual variables. The online marketing strategies, such as prior surveys, advertisements, and competition events, are just efforts in building up the relationships between governments and the citizens. Prior online surveys may help the government know better about citizens’ needs, attitudes, and perceptions toward the co-production programs and incorporate this understanding into the technological system design (Colasanti et al., 2018). Moreover, online advertisements and competition events may help the government attract multiple actors (not merely the citizens) with relevant skills, resources, expertise, and knowledge to engage in the co-production programs (Yuan & Gasco-Hernandez, 2021).

*Second, data collaboration is also an important cornerstone for performance value creation.* This is because data collaboration is often a necessary condition for generating service outcomes in digital co-production programs. For example, the “e-residency” program in Estonia which is built upon a connecting layer between decentralized government databases makes the investment service for foreign investors possible (Kattel et al., 2020). Another typical example is the “one-stop government portal.” For example, the “GOV.UK” digital platform where the websites of all government departments and many other agencies and public bodies in the UK are merged, and citizens and businesses can find all services through this one-stop portal (Weerakkody & Dhillon, 2008). Another example is the Austrian “no-stop shop service” through integrating data from multiple government departments and providing proactive services without any paper form; that is, a citizen only needs to give his or her consent and does not have to complete repetitive forms or perform any action to receive services (Scholta et al., 2019).

#### Citizens’ whole-life value may come from citizen–citizen interaction, reputational incentives, and undifferentiated treatment

*First, our reviewed articles suggest that the online interactions between citizens, including scoring, commenting, voting, and particularly answering others’ questions, are beneficial in generating whole-life value for individual citizens.* For example, in the digital communities in the Netherlands, citizens are allowed to communicate with each other; citizens answer specific questions that other citizens raise regarding public service affairs on the basis of their personal experiences and thus helping others to solve problems. During the process of mutual aid, citizens may develop their informal social network in the virtual communities, and more importantly, they may generate an enhanced self-efficacy (Zhou et al., 2014). In addition, the citizens who receive help from others will generate a feeling of empathy and gain emotional support from citizen peers. This whole-life value development is something that cannot be provided by the government.

*Second, reputational incentives may help citizens to form whole-life value.* For example, in the civic crowdfunding cases, de Crescenzo et al. (2021) show that the backers involved in civic crowdfunding campaigns usually expect a non-financial or an emotional reward since they are driven by a sense of engagement and belonging toward local territories. That means, the backs do not expect a tangible or material reward in exchange for their financial contribution (Tomczak & Brem, 2013). The backers involved in civic crowdfunding campaigns seem to act as donors, who see their return only in form of happiness because their funds are used for honorable causes. In addition, Brabham (2012) shows that, with the presence of reputational incentives, citizens have higher expectations that their opinions will have an influence when taking part in crowdsourcing initiatives. In this way, this type of initiative seems to lead to a higher level of perceived self-efficacy on the part of citizens, which has been highlighted as an important determinant for citizens to develop their characteristics.

*Third, undifferentiated treatment may positively influence the formation of citizens’ whole-life value.* In the “311” reporting system in the U.S., people of different ages, races, and incomes can use the digital system equally (Clark et al., 2020; Xu & Tang, 2020). People will never worry about how different the government will respond to their reporting problems simply because of their ages, races, or incomes. This equal treatment will bring about positive whole-life values to citizens and facilitate the citizens to form a value cognition of social equality.

#### Citizens’ capacity value can be created from the design factors of media richness and dialogic loop

*First, we find that media richness may bring about the capacity value for citizens.* Our reviewed articles suggest that, in emergent circumstances, rich government messages in media can enhance citizens’ capacity to release their uneasy mood and take proactive responding strategies toward crisis. For instance, in responding to the COVID-19 pandemic, the Chinese government published narrative messages through social media to provide the public with a social, emotional, and task-related context. These messages consist of a variety of cues that both convey information on the crisis and help the public resolve ambiguity, anxiety, and uncertainty (Ngai et al., 2020). Provided with such media-rich information, the public is more capable to engage in crisis response and has a relatively higher self-protection ability during the crisis.

*Second, a few studies reveal that the embedment of dialogic loop functions is beneficial for creating capacity value at the individual locus.* The logic underlying this causal link is that dialogic loops, including the use of hashtags, the “@” function, and the interrogative sentences may encourage citizens to interact with governments and during the interactions, the governments may guide and train citizens to respond to certain risky and dangerous circumstances, thus increasing the citizens’ capacity of dealing with dangers or crisis. For example, in the case of Hurricane Florence, Hampton and Virginia shared a list of supplies on Facebook and asked the citizens: “What’s on your Hurricane checklist?” and “Are we missing anything?”. Wukich (2021) has approved that such dialogic loops are helpful in fostering citizens’ self-rescue ability.

#### A variety of technological design factors contribute to the creation of administrative value

*First, online marketing factors may bring about government transparency and public engagement.* The main purpose of government efforts in conducting surveys, advertising, and holding competitions is to make the co-production programs open to the public and increase the public’s awareness and knowledge of the programs. These marketing strategies are thus beneficial for government transparency and public engagement. Our review indicates that when the public knows better what the co-production programs are about, the public is more inclined to engage with these programs (Dellaert, 2019). For example, Royo and Yetano (2015) have proven that all the citizen participants agree that these marketing actions lead to an improved government image and transparency, and some citizens who are originally reluctant to engage in government affairs are now more inclined to engage.

*Second, online/offline integration factors and government-to-citizen communication factors may lead to greater government accountability and trust.* For example, in the crowdsourcing case of urban planning in Barcelona, two offline workshops were held for citizens to review online opinions; during the workshops, facilitators from the government published the summarized opinions from the digital platform and let citizens in the workshops to further comment, score, and revise them. According to Satorras et al. (2020), citizens in this co-production program believed that the government is quite accountable and trustworthy because the government treats their online opinions very seriously and pays extra efforts to refine their proposals. *Moreover, the government-to-citizen communication factors, such as dialogic loops and mandatory message replies, can contribute to the value creation of government accountability and trust.* Our previously mentioned examples on crisis response have shown that the dialogic loops are the government efforts to remind/train citizens about how to prepare for the crisis and conduct self-rescue. Citizens in crisis circumstances are more likely to regard such government actions as accountable and willing to trust the government (Chen et al., 2020; Meltzer et al., 2018; Young et al., 2018). Mandatory message reply also plays such a role: when citizens receive government replies ever after they submit proposals online, they will generate a feeling that the government becomes responsible for their proposals (Buntaine et al., 2021; Sjoberg et al., 2017).

*Third, both incentivizing factors and EoU factors may result in more public engagement and stronger government decision legitimacy.* For instance, many studies have revealed that the bonus in civic hackathons, the acknowledgment of winners, and the “ease-of-use” interface design will trigger more citizens to engage with the digital co-production programs (Brabham, 2012; Leston-Bandeira, 2019; Moon, 2018). And scholars have recognized that the legitimacy of government decisions is generated based on citizen engagement and expression of opinions. The e-participatory budgeting case is an illustration of this regard. Legard and Goldfrank (2021) show that roughly two-thirds of online voters reported that they would not have voted in participatory budgeting if there had been no online option; and that the budget decisions gained substantial legitimacy by enhancing the inclusion and empowerment of citizens.

*Fourth, some EoU factors, including multilingual, non-professional language, and undifferentiated treatment may contribute to the creation of equality.* The logic underlying this causal link is that when the system is multilingual and designed with non-professional language, the citizens’ access to and use of the system will not be constrained by their demographical backgrounds and language conditions, thus obtaining an equal opportunity to use the system and engage in public services. In addition, when the system collects citizens’ private information as minimum as possible, the system will not recognize the demographical characteristics of citizens and thus treat them equally without any difference. The case of “311” citizen reporting system in the U.S. undoubtedly demonstrates this causal linkage (Clark et al., 2013; Wukich, 2021).

## Conclusions and future research agenda

IN this section, we first summarize our findings and then based on the review results we propose a future research agenda on digital co-production.

### A summary of findings

This review aims to identify the technological system design factors in digital co-production programs, the value elements created from these programs, and the potential relationships between the technological design factors and value creation. Our review results show that currently worldwide digital co-production programs apply seven design factors for their technological systems. These design factors encompass “ease-of-use” design, the presence of monetary and reputational incentives, and the government-to-citizen and citizen-to-citizen communication rules that make the system’s interface attractive and easy to use. Also, there are efforts of governments to conduct online marketing, integrate offline endeavors with online activities, and collaboration with external actors to share data, which are critical for background system construction and make the system more reliable for citizens’ usage. Besides, our review also finds that in digital co-production programs, five value categories can be identified, consisting of the short-term experiential value of individual citizens, the medium-term performance value of service systems, the long-term whole-life value of individual citizens, the capacity value of individual citizens, and the administrative value of governments.

Additionally, the review results show that the technological system design factors indeed play significant roles in facilitating value creation. First, for individual citizens, experiential value creation largely depends on the technological system’s ease-of-use design. The whole-life value creation is seldom seen, but it sporadically spreads in the technological systems that allow citizen-to-citizen interactions, and are designed with reputational incentives and undifferentiated treatment. The capacity value creation mainly exists in the cases of crisis response, and it can be facilitated by the design rules for government-to-citizen communication, such as media-rich information provision and the adoption of dialogical loops. For the governments, a variety of administrative values can be created, including greater engagement, legitimacy of decision-making, accountability, equality, transparency, and trust. Online marketing factors may bring about government transparency and engagement. Online/offline integration factors and government-citizen communication factors may lead to greater government accountability and trust. Both incentivizing factors and ease-of-use factors may result in more engagement and stronger government decision legitimacy. And some ease-of-use factors, including multilingual, non-professional language, and undifferentiated treatment may contribute to the creation of service equality. Regarding the service systems, the performance value creation relies on the efforts of the governments to build up good relationships with citizens and to open data-sharing channels with other governmental peers. Consequently, the online marketing strategies and data collaboration efforts play critical roles in ensuring the multi-actor systems of co-production programs function well.

### A future research agenda on (digital) co-production

Given these findings, what does a possible future research agenda look like?

First, this SLR confirms the point in the extant literature that co-production goes together with value creation. However, little efforts have been made regarding how to use “value creation” to evaluate co-production performance or outcome. Therefore, one possible future research direction might be value-based co-production evaluation. Our SLR reveals that value creation can happen at the individual (i.e., citizen) locus (e.g., experiential value, whole-life value, and capacity value), the service system locus (e.g., performance value), and the government locus (e.g., administrative value). Therefore, researchers might conduct surveys and/or interviews to examine how citizens, public decision-makers, and government administrators perceive the values they obtain from participating co-production programs. Researchers might also compare how different the values are created at the citizen, service system, and government loci in a single co-production program, considering that some co-production programs might only create values within government administration, while others contribute values to citizens. Furthermore, researchers could conduct cross-case studies to compare the levels of value creation in different co-production programs, and to investigate what barriers and drivers exist to block and enable value creation.

Second, this SLR indicates that technological system design factors can indeed bring about value creation in digital co-production programs. However, the extant studies in our records only examined the impacts of single technological design factors on value creation, few research has been done regarding what technological design factors, in combination, can lead to different levels of value creation. Therefore, we propose a future research direction that examines the mix and match of the technological design factors and their joint influence on value creation. In this direction, researchers are advised to conduct fuzzy-set Qualitative Comparative Analysis (QCA) on a small-N sample of digital co-production programs, to find out which technological design factors are necessary conditions for value creation, and which factors are sufficient ones. In addition, via this QCA approach, researchers might find different possible paths (i.e., different combinations of technological design factors) that lead to different levels of value creation. This has significant practical meanings to governmental organizations because not all governments have adequate technological capacities. For those with limited technological capacities, they might consider lower-level technological system design and just focus on specific types of value creation.

### Limitations of this SLR and suggestions on future review work

First, our review uses Loeffler’s “4-Co” model to identify the keywords for searching articles on digital co-production. However, digital co-production may take many forms which reside outside the “4-Co” model and may generate value elements that go beyond only individual, service system, and government loci. Therefore, our first suggestion for future SLR in this field is to supplement and expand the typologies of digital co-production and value elements with more empirical cases.

Second, in our review, we confine the co-production activities to the collaboration between governments and citizens; however, there is an increasing amount of literature that argues co-production does not merely refer to the joint efforts between governments and citizens, but also the collaboration between governments and private organizations or nongovernmental organizations. Therefore, our second suggestion for future SLR in this field is to expand the boundary of actors who may work together with governments through digital technologies and to examine what value may be created.

Third, our systematic review takes a static perspective, thus ignoring the dynamic change of the technological design factors in facilitating the creation of value elements, neither the change of value creation. However, the fact is that many digital co-production programs will be updated with new technologies or their technological systems will be redesigned with new functions or interfaces. This will bring about new design factors and possibly the creation of new value elements. Consequently, our suggestion for future SLR in this field would be about the dynamic mechanisms that lead various technological design factors to value creation.

## Supplementary Information

Below is the link to the electronic supplementary material.

Supplementary file1 (DOCX 82 KB)

## Footnotes

1 Or service providers and professionals mandated by or acting on behalf of governments.

2 Or service users and communities.
