# Supplementary material for: How does technological system design affect value creation? A systematic literature review of digital co-production
Source: Glob Public Policy Gov. 2022 Oct 28;2(4):400–26. doi: 10.1007/s43508-022-00051-0 (PMC9628496; doi:10.1007/s43508-022-00051-0)
Supplement: Supplementary file 1 — Supplementary file1 (DOCX 82 KB) [file 43508_2022_51_MOESM1_ESM.docx]

Appendix 1. Descriptive information of sample articles (articles listed in a chronological order)

| Article titles and hyperlinks | Authors | Publication years | Publication journals | Countries under analysis | Government layers | Digital technology used |
| --- | --- | --- | --- | --- | --- | --- |
| An assessment of public participation GIS and web 2.0 technologies in urban planning practice in Canela, Brazil  <https://doi.org/10.1016/j.cities.2009.11.008> | Bugs, G.;  Granell, C.;  Fonts, O.;  Huerta, J.;  Painho, M. | 2010 | Cities | Brazil | Local government | Nongovernmental website |
| A virtual globe-based 3D visualization and interactive framework for public participation in urban planning processes  <https://doi.org/10.1016/j.compenvurbsys.2009.12.001> | Wu, HY.;  He, ZW.;  Gong, JY. | 2010 | Computers Environment and Urban Systems | China | Local government | Nongovernmental website |
| Networked coproduction of public services in virtual communities: from a government-centric to a community approach to public service support  <https://doi.org/10.1111/j.1540-6210.2011.02391.x> | Meijer, A J. | 2011 | Public Administration Review | The Netherlands | Local government | Government portal |
| Motivations for participation in a crowdsourcing application to improve public engagement in transit planning  <http://dx.doi.org/10.1080/00909882.2012.693940> | Brabham, D C. | 2012 | Journal of Applied Communication Research | USA | Local government | Government portal |
| Implementing open innovation in the public sector: the case of challenge.gov  <https://doi.org/10.1111/puar.12141> | Mergel, I.;  Desouza, K C. | 2013 | Public Administration Review | USA | Central government | Government portal |
| Coproduction of government services and the new information technology: investigating the distributional biases  <https://doi.org/10.1111/puar.12092> | Clark, B Y.;  Brudney, J.;  Jang, S G. | 2013 | Public Administration Review | USA | Local government | Government portal;  APP |
| Digital serious game for urban planning: B3-design your marketplace!  <https://doi.org/10.1068/b39032> | Poplin, A. | 2014 | Environment and Planning B-Planning &Design | Germany | Local government | Nongovernmental website |
| New media and the coproduction of safety: an empirical analysis of Dutch practices  <https://doi.org/10.1177/0275074012455843> | Meijer, A J. | 2014 | The American Review of Public Administration | The Netherlands | Local government | Nongovernmental website |
| Crowdsourcing as a tool for e-participation: two experiences regarding CO_2_ emissions at municipal level  <https://doi.org/10.1007/s10660-015-9183-6> | Royo, S.;  Yetano, A. | 2015 | Electronic Commerce Research | Spain | Local government | Government portal |
| Political participation via social media: a case study of deliberative quality in the public online budgeting process of Frankfurt/Main, Germany 2013  <https://doi.org/10.1007/s10209-014-0353-4> | Pieper, A K.;  Pieper, M. | 2015 | Universal Access in the Information Society | Germany | Local government | Government portal |
| The contradictory effects in efficiency and citizens’ participation when employing Geo-ICT apps within local government  <http://dx.doi.org/10.1080/03003930.2014.937001> | Kurniawan, M.;  de Vries, W T. | 2015 | Local Government Studies | The Netherlands | Local government | Nongovernmental website;  APP |
| Exploring online engagement in public policy consultation: the crowd or the few?  <http://dx.doi.org/10.1111/1467-8500.12209> | Liu, H K. | 2017 | Australian Journal of Public Administration | USA;  Australia | Local government | Nongovernmental website |
| The effect of bureaucratic responsiveness on citizen participation  <https://doi.org/10.1111/puar.12697> | Sjoberg, F M.;  Mellon, J.;  Peixoto, T. | 2017 | Public Administration Review | UK | Local government | Nongovernmental website;  APP |
| Uncharted territoriality in coproduction: the motivations for 311 reporting  <https://doi.org/10.1093/jopart/muw046> | O’Brien, D T.;  Offenhuber, D.;  Baldwin-Philippi, J.;  Sands, M.;  Gordon, E. | 2017 | Journal of Public Administration Research and Theory | USA | Local government | APP |
| Open innovation in the public sector: drivers and barriers for the adoption of Challenge.gov  <https://doi.org/10.1080/14719037.2017.1320044> | Mergel, I. | 2018 | Public Management Review | USA | Central government | Government portal |
| Evolution of co-production in the information age: crowdsourcing as a model of web-based co-production in Korea  <https://doi.org/10.1080/14494035.2017.1376475> | Moon, M J. | 2018 | Policy and Society | Korea | Local government | Nongovernmental website;  APP |
| Keep them engaged: Romanian county inspectorates for emergency situations’ Facebook usage for disaster risk communication and beyond  <https://doi.org/10.3390/su10051411> | Meltzer, M.;  Stefanescu, L.;  Ozunu, A. | 2018 | Sustainability | Romania | Local government | Facebook |
| #Engagement: use of Twitter chats to construct nominal participatory spaces during health crises  <https://doi.org/10.1080/1369118X.2017.1301518> | Young, R.;  Tully, M.;  Dalrymple, K E. | 2018 | Information Communication& Society | USA | Central government | Twitter |
| Higher education and stakeholders’ donations: successful civic crowdfunding in an Italian university  <https://doi.org/10.1080/09540962.2018.1449471> | Colasanti, N.;  Frondizi, R.;  Meneguzzo, M. | 2018 | Public Money & Management | Italy | Local government | Nongovernmental website |
| Citizen engagement and co-production of e-government services in China  <https://doi.org/10.1080/23812346.2019.1705052> | Ma, L.;  Wu, X. | 2019 | Journal of Chinese Governance | China | Central government | Government portal |
| Parliamentary petitions and public engagement: an empirical analysis of the role of e-petitions  <https://doi.org/10.1332/030557319X15579230420117> | Leston-Bandeira, C. | 2019 | Policy and Politics | UK | Central government | Government portal |
| Does mapping improve public participation? Exploring the pros and cons of using public participation GIS in urban planning practices  <https://doi.org/10.1016/j.landurbplan.2019.02.019> | Kahila-Tani, M.;  Kytta, M.;  Geertman, S. | 2019 | Landscape and Urban Planning | Finland;  USA;  Germany | Local government | Nongovernmental website |
| Evaluating the scalability of public participation in urban land use planning: a comparison of Geoweb methods with face-to-face meetings  <https://doi.org/10.1177/2399808317719709> | Jankowski, P.;  Czepkiewicz, M.;  Mlodkowski, M.;  Zwolinski, Z.;  Wojcicki, M. | 2019 | Environment and Planning B-Urban Analytics and City Science | Poland | Local government | Nongovernmental website |
| Urban planning, public participation and digital technology: app development as a method of generating citizen involvement in local planning processes  <https://doi.org/10.1177/2399808317712515> | Wilson, A.;  Tewdwr-Jones, M.;  Comber, R. | 2019 | Environment and Planning B-Urban Analytics and City Science | UK | Local government | APP |
| Crowdsourcing Bike Share Station Locations  <https://doi.org/10.1080/01944363.2018.1476174> | Griffin, G P.;  Jiao, J. | 2019 | Journal of the American Planning Association | USA | Local government | Government portal |
| Collaborative innovation and human-machine networks  <https://doi.org/10.1080/14719037.2019.1645873> | Kattel, R.;  Lember, V.;  Tonurist, P. | 2020 | Public Management Review | Estonia | Central government | Government portal |
| The public’s acceptance of and intention to use ICTs when participating in urban planning processes  <https://doi.org/10.1080/10630732.2020.1852816> | Li, W S.;  Feng, T.;  Timmermans, H J P.;  Zhang, M. | 2020 | Journal of Urban Technology | China | Local government | Government portal;  Weibo |
| Co-production of urban climate planning: insights from the Barcelona climate plan  <https://doi.org/10.1016/j.cities.2020.102887> | Satorras, M.;  Ruiz-Mallen, I.;  Monterde, A.;  March, H. | 2020 | Cities | Spain | Local government | Government portal |
| Developing a multi-level organization-public dialogic communication framework to assess social media-mediated disaster communication and engagement outcomes  <https://doi.org/10.1016/j.pubrev.2020.101949> | Liu, W L.;  Xu, W A.;  Tsai, J Y. | 2020 | Public Relations Review | USA | Local government | Facebook |
| Unpacking the black box: how to promote citizen engagement through government social media during the COVID-19 crisis  <https://doi.org/10.1016/j.chb.2020.106380> | Chen, Q.;  Min, C.;  Zhang, W.;  Wang, G.;  Ma, X Y.;  Evans, R. | 2020 | Computers in Human Behavior | China | Central government | Weibo |
| Grappling with the COVID-19 health crisis: content analysis of communication strategies and their effects on public engagement on social media  <http://www.jmir.org/2020/8/e21360/> | Ngai, C S B.;  Singh, R G.;  Lu, W Z.;  Koon, A C. | 2020 | Journal of Medical Internet Research | China | Central government | Weibo |
| Is crowdsourcing a reliable method for mass data acquisition? The case of COVID-19 spread in Greece during spring 2020  <https://doi.org/10.3390/ijgi9100605> | Antoniou, V.;  Vassilakis, E.;  Hatzaki, M. | 2020 | ISPRS International Journal of Geo-Information | Greece | Local government | Nongovernmental website |
| Participatory surveillance based on crowdsourcing during the Rio 2016 Olympic Games using the Guardians of health platform: descriptive study  <https://doi.org/10.2196/16119> | Neto, OL.;  Cruz, O.;  Albuquerque, J.;  de Sousa, MN.;  Smolinski, M.;  Cesse, EAP.;  Libel, M.;  de Souza, WV. | 2020 | JMIR Public Health and Surveillance | Brazil | Local government | APP |
| Laboratories for news? Experimenting with journalism hackathons  <https://doi.org/10.1177/1464884917737213> | Boyles, J L. | 2020 | Journalism | USA;  Australia;  UK;  The Netherlands;  Norway;  Colombia;  Costa Rica | Local government | Government portal |
| Closing the gap or widening the divide: the impacts of technology-enabled coproduction on equity in public service delivery  <https://doi.org/10.1111/puar.13222> | Xu, C K.;  Tang, T. | 2020 | Public Administration Review | USA | Local government | APP |
| IT services and crowdsourcing in support of the Hellenic cadastre: advanced citizen participation and crowdsourcing in the official property registration process  <https://doi.org/10.3390/ijgi9040190> | Mourafetis, G.;  Potsiou, C. | 2020 | ISPRS International Journal of Geo-Information | Greece | Local government | Government portal |
| Witnessing a disaster: public use of digital technologies in the 2015 south Indian floods  <https://doi.org/10.1080/21670811.2019.1636693> | Paul, S.;  Sosale, S. | 2020 | Digital Journalism | India | Local government | Nongovernmental website;  Facebook;  Twitter |
| Do advanced information technologies produce equitable government responses in coproduction: an examination of 311 systems in 15 US cities  <https://doi.org/10.1177/0275074019894564> | Clark, B Y.;  Brudney, J L.;  Jang, S G.;  Davy, B. | 2020 | American Review of Public Administration | USA | Local government | APP |
| Open innovation in the public sector: creating public value through civic hackathons  <https://doi.org/10.1080/14719037.2019.1695884> | Yuan, Q L.;  Gasco-Hernandez, M. | 2021 | Public Management Review | USA | Local government | Government portal |
| Reuse of open data in Quebec: from economic development to government transparency  <https://doi.org/10.1177/0020852319884628> | Boudreau, C. | 2021 | International Review of Administrative Science | Canada | Local government | Government portal |
| Civic crowdfunding: a new opportunity for local governments  <https://doi.org/10.1016/j.jbusres.2020.10.021> | De Crescenzo, V.;  Botella-Carrubi, D.;  Garcia, M R. | 2021 | Journal of Business Research | Italy | Local government | Government portal |
| Increasing citizen participation in e-participatory budgeting processes  <https://doi.org/10.1080/19331681.2020.1821421> | Mærøe, A R.;  Norta, A.;  Tsap, V.;  Pappel, I. | 2021 | Journal of Information Technology & Politics | Estonia | Local government | Government portal |
| Transforming urban planning processes and outcomes through creative methods  <https://doi.org/10.1007/s13280-020-01436-3> | Cinderby, S.;  de Bruin, A.;  Cambridge, H.;  Muhoza, C.;  Ngabirano, A. | 2021 | AMBIO | Kenya;  Uganda | Local government | Government portal |
| Evaluation of mobile-based public participation in China’s urban planning: case study of the PinStreet platform  <https://doi.org/10.1016/j.cities.2020.102993> | Wang, X.;  Chen, Y L.;  Han, Z Y.;  Yao, X Y.;  Gu, P Q.;  Jiang, Y. | 2021 | Cities | China | Local government | APP |
| The systemic turn and participatory budgeting: the case of Rio Grande do Sul  <https://doi.org/10.1017/S0022216X20000954> | Legard, S.;  Goldfrank, B. | 2021 | Journal of Latin American Studies | Brazil | Central government | Government portal;  Facebook;  Twitter |
| Promoting public engagement during the COVID-19 crisis: how effective is the Wuhan local government’s information release?  <https://doi.org/10.3390/ijerph18010118> | Yang, Y.;  Deng, W.;  Zhang, Y.;  Mao, Z J. | 2021 | International Journal of Environmental Research and Public Health | China | Local government | Weibo |
| Government social media engagement strategies and public roles  <https://doi.org/10.1080/15309576.2020.1851266> | Wukich, C. | 2021 | Public Performance & Management Review | USA | Local government | Facebook |
| Organizing innovation contests for public procurement of innovation - a case study of smart city hackathons in Tampere, Finland  <https://doi.org/10.1080/09654313.2021.1894097> | Pihlajamaa, M.;  Merisalo, M. | 2021 | European Planning Studies | Finland | Local government | Government portal |
| Open innovation in the face of the COVID-19 grand challenge: insights from the Pan-European hackathon “EUvsVirus”  <https://doi.org/10.1111/radm.12456> | Bertello, A.;  Bogers, M L A M.;  De Bernardi, P. | 2021 | R&D Management | European Union | International level | Government portal |
| Escaping the disengagement dilemma: two field experiments on motivating citizens to report on public services  <https://doi.org/10.1017/S0007123419000322> | Buntaine, M T.;  Nielson, D L.;  Skaggs, J T. | 2021 | British Journal of Political Science | Uganda | Local government | APP |
| Harnessing the power of dialogue: examining the impact of facebook content on citizens’ engagement  <https://doi.org/10.1080/03003930.2020.1870958> | Lappas, G.;  Triantafillidou, A.;  Kani, A. | 2021 | Local Government Studies | Greece | Local government | Facebook |
| Assessing impacts of PPGIS on urban land use planning: evidence from Finland and Poland  <https://doi.org/10.1080/09654313.2021.1882393> | Jankowski, P.;  Forss, K.;  Czepkiewicz, M.;  Saarikoski, H.;  Kahila, M. | 2021 | European Planning Studies | Poland;  Finland | Local government | APP |

**Appendix 2. The coding process**

| Articles | Technological system design factor | | | Value creation | | |
| --- | --- | --- | --- | --- | --- | --- |
|  | Textual extraction | Open coding | Axial coding | Textual extraction | Open coding | Axial coding |
| Bugs et al. (2010) | “The commenting tool allows users to enter their opinion by selecting an icon whose color identifies a planning topic and placing it on the map.” (p.176) | Score, comment, like and vote citizen messages | Citizen-citizen interaction factors | “Opinion exchanges are fostered by allowing users to inspect comments for the same thread introduced by other participants” (p.176) Citizens can express their preferences in the comments section and find opinions with similar points of view to comment on, gaining a sense of identity. | Self-efficacy | Whole-life value |
| Wu et al. (2010) | “The virtual globe-based design, using AJAX (Asynchronous JavaScript and XML) technology, allows end users to browse the virtual urban planning environment by various operations.” (p.294) | Visualization | Ease-of-use factors | “AJAX makes possible a totally new end user experience: end users smoothly manipulate resources from the Internet as if they are operating on their local computers.” (p.293) | Good participation experience | Experiential value |
| Meijer (2011) | “The forum also provides another channel of communication with the agency, as moderators from the agency also respond to questions and comments from citizens.” (p.602) | Little helper | Ease-of-use factors | “Moderators respond quickly and show respect for the users. Moderators provide valuable answers to questions that are not answered by other users.” (p.603) | User satisfaction on service | Experiential value |
|  | “On this website to enable citizens to ask each other questions and discuss issues related to jobs and unemployment.” (p.602) | Score, comment, like and vote citizen messages | Citizen-citizen interaction factors | “Enabling citizens to exchange experiences and answer each other’s questions, which providing emotional support.” (p.600) | Self-efficacy | Whole-life value |
| Brabham (2012) | “Participants could register a free account by completing a registration process that included questions about past bus ridership, past public participation in urban planning issues, and demographic information.” (p.309) | Convenient registration or login | Ease-of-use factors | “Participants repeatedly mentioned perceived low barriers to entry and appealing Web site design as reasons they were motivated to visit and participate on the site.” (p.319) | User satisfaction on service | Experiential value |
|  | “The only reward offered by the competition to the winners was acknowledgment of their win in the form of a press release to media and announcement on the Web site itself. (p.310) | Reputational incentives | Incentivizing factors | “If my designs get real, and if people use that, I feel so happy. And this thought is getting me so excited.” (p.320) | Self-efficacy | Whole-life value |
|  | “Registered participants could also comment and cast a 1-5 point vote for each submitted design in the competition.” (p.309) | Score, comment, like and vote citizen messages | Citizen-citizen interaction factors | “To contribute to a collaborative effort.” (p.320) |  |  |
| Mergel & Desouza (2013) | “The Office of Citizen Services and Innovative Technologies supports the agency by promoting competitions to the general public through traditional channels, such as press releases, but also by harnessing social media channels.” (p.884) | Online advertisement | Online marketing factors | “The office uses social media to reach out to potential participants.” (p.887) | Engagement | Administrative value |
|  |  |  |  | “Citizens provide solutions but also review and evaluate solutions, vote on solutions, and even get involved in the implementation of solutions and subsequent evaluation of new policies or other types of public sector innovations.” (p.884) | Innovative solution | Performance value |
| Clark et al. (2013) | Identity information such as race and age are not required for registration and use. | Undifferentiated treatment | Ease-of-use factors | “We have little cause for concern that 311 systems may benefit one group over another based on race.” (p.688) | Value cognition of social equality | Whole-life value |
| Poplin (2014) | “A ‘little helper’ is designed to interact with the player and offers suggestions on how to use different functionalities of the game.” (p.501) | Little helper | Ease-of-use factors | “It is included in the game to encourage and guide the player, and to communicate with her while navigating through the game environment.” (p.501) | Good participation experience | Experiential value |
|  | “The players can give up to five stars to other players’ design.” (p.501) | Score, comment, like and vote citizen messages | Citizen-citizen interaction factors | “The number of stars for each design is calculated as the mean value of all votes contributed.” (p.501) Citizens who get higher scores in the rating will feel that their plans and ideas are recognized by the majority, then generate a sense of fulfillment. | Self-efficacy | Whole-life value |
| Meijer (2014) | “Citizens are targeted on the basis of their geographical characteristics.” (p.27) | GIS | Ease-of-use factors | According to citizens' GIS information, when a crime or disappearance occurs, the nearby residents will receive real-time contact from the police to assist in providing relevant information. | Good participation experience | Experiential value |
| Royo & Yetano (2015) | “Several steps were taken in each city to give publicity to the crowdsourcing initiative, including press releases; e-mails to neighborhood associations, consumer associations and major organizations working in the field of the environment; and information sent by e-mail to staff and students of the public universities of the respective regions.” (p.331) | Online advertisement | Online marketing factors | “In both cases, the platform remained open for a period of 10 weeks. During this time, 231 and 223 valid responses were received.” (p.331) | Engagement | Administrative value |
|  |  |  |  | “To let citizens propose other measures that could be adopted by the local government in order to reduce CO_2_ emissions.” (p.331) | Feasible solution | Performance value |
| Pieper & Pieper (2015) | “The discussion took a momentum that threatened to break the above-mentioned rules of Netiquette, which is why straightway moderators intervened in the communication process.” (p.498) | Little helper | Ease-of-use factors | “They ensured compliance with discursive conditions of the so-called Netiquette. The moderators also answered on issues of content or procedure criticism.” (p.497) | User satisfaction on service | Experiential value |
|  | “The citizens had the possibility to submit suggestions in particular online, but also traditionally by mail and phone. Budgetary administration transmitted those analog proposals into online versions.” (p.496) | Mix use of online/offline channels | Online/offline integration factors | “Citizen budgeting suppose a certain degree in direct involvement and reinforced discussion motivation of the citizens.” (p.495) | Engagement  Legitimacy | Administrative value |
| Kurniawan & de Vries (2015) | “In this case, they can use the Verbeterdebuurt on their smart-phone to take a picture and pinpoint the location of the idea.” (p.128) | GIS | Ease-of-use factors | “All reported problems appear on a map” (p.126), which implies that citizens can be guaranteed that their reports have been received by the system. | Good participation experience | Experiential value |
| Liu (2017) | “To identify the most useful ideas, the platform adopted a rating system. Each entry was given a score by summing the totals for thumbs-up (+1) and for thumbs-down (–1).” (p.37) | Score, comment, like and vote citizen messages | Citizen-citizen interaction factors | “Ideas and discussions generated from the Open Government Dialogue subsequently formed the basis for the Open Government Policy launched in 2009.” (p.37) This indicates that citizens’ opinions have the opportunity to be included in the official government agenda, which makes citizens feel empowered. | Self-efficacy | Whole-life value |
| Sjoberg et al. (2017) | “A user enters a U.K. postcode or street name or uses the ‘locate automatically’ function. A map is then shown covering the area of interest. The user clicks the map to indicate the specific location of the problem and enters a subject line, a short description, a category” (p.343) | Visualization and GIS | Ease-of-use factors | “Report submission is a simple process taking, on average, only a few minutes to complete.” (p.343) | Good participation experience | Experiential value |
|  | “Local authorities can respond to these reports through the platform by indicating when the problem is fixed.” (p.343) | Mandatory message reply | Government-citizen communication factors | “The short-term model suggests that users whose first reported problem was fixed are 57 percent more likely to send at least one more report.” (p.349) | Engagement  Legitimacy  Accountability | Administrative value |
| O’Brien et al. (2017) | “Constituents can register with the Boston 311 system, creating an account that archives and tracks all of his or her requests.” (p.324) | Personal webpage | Ease-of-use factors | “A 311 user account compiles all reports made by an individual.” (p.325) | User satisfaction on service | Experiential value |
|  |  |  |  | This system can track the progress of your application. | Transparency | Administrative value |
| Mergel (2018) | “US federal government agencies are using a shared online platform called Challenge.gov to post their problem statements and collect ideas from citizens.” (p.726) | Online competition events | Online marketing factors | “The broad participation of problem solvers who were never in touch with government showed them the potential of existing slack capacity among external problem solvers.” (p.735) | Engagement | Administrative value |
|  |  |  |  | “I think there is just an overall embrace in general about looking at new ways of doing work and having outside people help us with the work that we do.” (p.734) | Innovative solution | Performance value |
| Moon (2018) | “To promote active citizen participation in making policy suggestions, the SMG offers small cash awards equivalent to USD $50 and $200 for those suggestions that are partially or fully adopted by the government, respectively.” (p.302) | Monetary reward | Incentivizing factors | “To promote active citizen participation in making policy suggestions, the SMG offers small cash awards.” (p.302) | Engagement | Administrative value |
|  | “Citizens can access the portal via the web or an app and propose policy suggestions, which are later reviewed, assessed and elaborated by government officials. The review and evaluation are often conducted in offline meetings in which government officials, policy experts and citizens refine the suggestion.” (p.302) | Mix use of online/offline channels | Online/offline integration factors | “This crowdsourcing co-design approach seeks to invite citizen participation in solving public policy problems, opening the doors wide to citizens in the design and planning stages by allowing them to offer any suggestion or solution for policies and public services presented by governments.” (p.302) | Engagement  Legitimacy | Administrative value |
| Meltzer et al. (2018) | Use of photos and videos. | Media richness | Government-citizen communication factors | Citizens’ ability to cope with crisis situations. | Crisis response capacity | Capacity value |
|  |  |  |  | “Photos and videos have a positive effect on stakeholder interaction.” (p.15) | Engagement | Administrative value |
| Young et al. (2018) | Use of #. | Dialogic loop | Government-citizen communication factors | Citizens’ ability to cope with crisis situations. | Crisis response capacity | Capacity value |
|  |  |  |  | “This strategic use of a hashtag creates a bounded space of participation around topics and issues in which the hashtag is the organizing principle for conversations.” (p.502) | Accountability  Engagement | Administrative value |
| Colasanti et al. (2018) | “Sent an initial survey to a sample of 5,800 internal stakeholders in the University of Rome ‘Tor Vergata’.” (p.284) | Prior online surveys | Online marketing factors | “The preliminary survey showed a strong interest in the project, which led to setting up and launching the campaign.” (p.284) | Engagement | Administrative value |
| Ma & Xu (2019) | “All certified government websites are required to embed the IFMGW (I find mistakes for government websites) button at their bottom.” (p.6) | Online advertisement | Online marketing factors | “Any user who find errors and mistakes can click the button to report to the platform. (p.6) This setting greatly facilitates citizens' online participation. | Engagement | Administrative value |
|  |  |  |  | “To complete the identification and treatment of website errors in cooperation with the public.” (p.6) If there are no citizens involved in this work, it will consume a lot of time and energy of government officials. | Efficiency | Performance value |
| Leston-Bandeira (2019) | “The e-petitions site is very accessible. Contrary to other systems, there is no need for registration and a handful of clicks accompanied by 380 characters are enough to submit an e-petition.” (p.424) | Convenient registration or login. | Ease-of-use factors | “As one of our interviewees said ‘it is dangerously easy to submit a petition, which can be done in a few minutes’.” (p.424) | User satisfaction on service | Experiential value |
|  | “Each e-petition has its own webpage clearly listing all of the relevant actions taken by government and parliament.” (p.425) | Personal webpage | Ease-of-use factors | “Each e-petition has its own webpage clearly listing all of the relevant actions taken by government and parliament.” (p.425) |  |  |
|  |  |  |  | “The system is also considerably transparent.” (p.425) | Transparency | Administrative value |
|  | “The response from government departments to e-petitions reaching 10,000 signatures, within a deadline of 21 days.” (p.423) | Mandatory message reply | Government-citizen communication factors | “By simply coming across as listening and responding to queries, it performed a legitimacy role, whereby people’s trust in the overall system is reinforced.” (p.427) | Engagement  Legitimacy  Accountability |  |
| Kahila-Tani et al. (2019) | “Being able to easily demonstrate current plans and potential outcomes of a project/investment overlaid on the map.” (p.49) | Visualization | Ease-of-use factors | “Respondent accessibility is promoted by the usability, visual appearance and scaling of the PPGIS tool from mobile devices to laptops.” (p.50) | Good participation experience | Experiential value |
|  | “In 19% of cases, two language version were available and in 2% three languages.” (p.47) | Multilingual | Ease-of-use factors | Multilingual design helps to enhance the user experience. |  |  |
|  |  |  |  | Multilingual design can attract more non-native English speakers to use the APP. | Engagement  Equality | Administrative value |
| Jankowski et al. (2019) | “One of the reasons for the high credibility of the online consultations was the publication of their results in a report.” (p.526) | Mandatory message reply | Government-citizen communication factors | “It was the first such document ever in the planning practice of the Municipal Planning Office. The lack of similar reports has been seen as a major drawback of traditional public meetings.” (p.526) | Engagement  Legitimacy  Accountability | Administrative value |
| Wilson et al. (2019) | “When user have entered an area within which a change was proposed, they would receive a notification.” (p.288) | Push notification | Ease-of-use factors | “The notification contained an easily identifiable ChangeExplorer icon, allowing users to quickly understand that the app was seeking their comments.” (p.292) | Good participation experience | Experiential value |
|  |  |  |  | “Digital wearables are easier readability and fewer missed notifications than smart phones.” (p.517) | Engagement | Administrative value |
|  | “To avoid a problem with comments being too general ChangeExplorer used categories to guide the user to planning related comments.” (p.293) | Score, comment, like and vote citizen messages | Citizen-citizen interaction factors | “The intention of the platform was to allow for open-ended dialogues around place. The categories aided the sorting of these comments into different discussions that were taking place.” (p.295) Citizens can express their preferences in the comments section and find opinions with similar points of view to comment on, gaining a sense of identity. | Self-efficacy | Whole-life value |
| Griffin & Jiao (2019) | “The platforms also incorporated the ability for users to like and comment on others’ suggestion.” (p.40) | Score, comment, like and vote citizen messages | Citizen-citizen interaction factors | Participants provided many suggestions and offered insights and local knowledge in their written defense or description of suggested sites. (p.43) Citizens are empowered to contribute their knowledge and proposals, and may ultimately influence government choices and arrangements for site planning. | Self-efficacy | Whole-life value |
|  | “Formal planning efforts for Citi Bike included in-person public meetings, websites with an interactive PPGIS platform allowing users to suggest bike share station locations, and GIS analysis by planners on optimal bike share station locations. Planners then combined the locations suggested on paper maps at in-person meetings with the PPGIS results.” (p.40) | Mix use of online/offline channels | Online/offline integration factors | “Our analysis suggests that such participatory mechanisms have great promise for creating genuine co-production of planning knowledge and insights. Participants provided many suggestions and offered insights and local knowledge in their written defense or description of suggested sites.” (p.45) | Engagement  Legitimacy | Administrative value |
| Kattel et al. (2020) | “The electronic ID card and secure data exchange architecture as a connecting layer between decentralized databases, e-ID for identification, played a crucial role, as their high acceptance, transparency and reliability enabled effective collaboration and rapid implementation in both cases.” (p.1664) | Data collaboration between government departments | Data collaboration factors | “The program has generated 14.4 M EUR of direct and indirect income for the government.” (p,1665) | Efficiency | Performance value |
| Li et al. (2020) | “Planning authorities need to provide explanations for the adoption and rejection of opinions collected from the public.” (p.59) | Mandatory message reply | Government-citizen communication factors | “To ensure widespread participation, ICT technologies are becoming important tools that enable citizens to participate in urban planning. Social media platforms are becoming easy channels where individualized opinions can be expressed. ICT technology and platforms provide a convenient, open, transparent, and interactive dialogue for the general public.” (p.70) | Engagement  Legitimacy  Accountability | Administrative value |
| Satorras et al. (2020) | “All of the 110 compiled proposals could be commented and voted on the same platform by users.” (p.7) | Score, comment, like and vote citizen messages | Citizen-citizen interaction factors | “Less than two thirds of these proposals (69/110; 63%) were included identically or slightly transformed in the final Climate Plan. ” (p.7) This indicates that citizens’ opinions have the opportunity to be adopted by the government, which makes citizens feel empowered. | Self-efficacy | Whole-life value |
|  | “Two offline workshops were held for organizations and citizens to solicit opinions, facilitators then summarized and published them in the digital platform, where users could still comment and value them.” (p.6) | Mix use of online/offline channels | Online/offline integration factors | “Thus, the platform channeled a large part of lay citizens participation.” (p.7) | Engagement  Legitimacy | Administrative value |
| Liu et al. (2020) | “The presence of a dialogic loop was indicated by phrases that invited the public to access information provided, contacted the organizations, or contributed to disaster-relief activities.” (p.5) | Dialogic loop | Government-citizen communication factors | Citizens’ ability to cope with crisis situations. | Crisis response capacity | Capacity value |
|  |  |  |  | “Dialogic loop was significantly associated with more shares that a post received.” (p.6)  “The use of richer media such as videos was significantly associated with more likes, comment.” (p.6) | Accountability  Engagement | Administrative value |
|  | Use of video. | Media richness |  |  |  |  |
| Chen et al. (2020) | “Five items are used including ‘the use of hashtags’, ‘the provision of surveys or votes for users to express opinions’, ‘the use of @ function’, ‘responding to a question’, and ‘posting a question’.” (p.6) | Dialogic loop | Government-citizen communication factors | Citizens’ ability to cope with crisis situations. | Crisis response capacity | Capacity value |
|  |  |  |  | “The IRR value showed that a one-unit increase in the level of dialogic loop would lead to an increase in the level of CEGSM (composed of the number of likes, reposts, and comments) by a factor of 1.35.” (p.7) | Accountability  Engagement | Administrative value |
| Ngai et al. (2020) | Use of narrative posts. | Media richness | Government-citizen communication factors | Citizens’ ability to cope with crisis situations. | Crisis response capacity | Capacity value |
|  |  |  |  | “Narrative posts generated significantly more shares than nonnarrative posts.” (p.7) | Engagement | Administrative value |
| Antoniou et al. (2020) | “Regarding participants’ personal information, only the location and the selected answer were recorded, along identification number. No other information was recorded that could disclose any personal information of the participants.” (p.5) | Privacy protection | Ease-of-use factors | Minimal user data collection is conducive to creating a good user experience. | Good participation experience | Experiential value |
|  |  |  |  | Minimizing data collection helps protect user privacy. | Trust | Administrative value |
| Neto et al. (2020) | The whole app was translated into seven languages: English, Spanish, Portuguese, French, Arabic, Chinese, and Russian. (p.2) | Multilingual | Ease-of-use factors | Multilingual design helps to enhance the user experience. | User satisfaction on service | Experiential value |
|  |  |  |  | Multilingual design can attract more non-native English speakers to use the APP. | Engagement  Equality | Administrative value |
| Boyles (2020) | “Hackathon organizers offer prizes, such as free software and gift cards.” (p.1345) | Monetary reward | Incentivizing factors | “Hackathon organizers lure participation from the public through offering prizes.” (p.1345) | Engagement | Administrative value |
|  | “Hack event organizers have taken to labeling their events as data dives, design-a-thons, code gatherings, design sprints, or maker fests.” (p.1345) | Non-professional language | Ease-of-use factors | Non-professional language allows citizens to understand the system and the service in a better way. | Good participation experience | Experiential value |
| Xu & Tang (2020) | “The restoration process did not favor or bias against any demographic group.” (p.970) | Undifferentiated treatment | Ease-of-use factors | “E-governance technologies provide ‘digital capital’ to the historically disadvantaged groups, which helps narrow and even close the equity gap in service delivery.” (p.971)  “Historically disadvantaged groups may greatly benefit from 311-based coproduction and that technology-enabled coproduction can help narrow or even close the equity gap in service delivery.” (p.970) | Value cognition of social equality | Whole-life value |
| Mourafetis &  Potsiou (2020) | “The Cadastral Declaration Web Site (CDWS) utilizes the TAXIS authentication system, the system that keeps the Greek citizens’ records for taxation purposes. Within TAXIS, each citizen has a unique identification number, which identifies him/her as a person.” (p.8) | Convenient registration or login. | Ease-of-use factors | Citizens need only unique identification number and no other information to log into the system. | Good participation experience | Experiential value |
|  |  | Data collaboration between government departments | Data collaboration factors | “Using the TAXIS credentials, the Cadastral Agency can directly identify the person without any further need for input.” (p.8) | Efficiency | Performance value |
| Paul & Sosale (2020) | “The collective also shared information with traditional news media outlets and government rescue teams. Twitter India shared the link to the collective’s website, and mainstream English-language newspaper, The Hindu, hosted a link to the collective on its website.” (p.20) | Data collaboration between government and NPOs | Data collaboration factors | “Mobile phones and social media enabled citizen journalists to gather, verify, and relay news and thereby helped in building the collective and coordinating rescue operations.” (p.27) Information sharing greatly saves the government’s manpower and time costs in searching for disaster relief information. | Efficiency | Performance value |
| Clark et al. (2020) | “The responses by governments are not systematically biased against traditionally disadvantaged groups.” (p.316) | Undifferentiated treatment | Ease-of-use factors | “Systematic biases are not built into technologically enabled coproduction and the ways governments are using it to allocate their efforts to deal with problems in city neighborhoods.” (p.324)  “This article provides evidence that largely shows no systematic biases in how local governments are providing services in neighborhoods of color, poor neighborhoods or neighborhoods with lower levels of educational attainment.” (p.325) | Value cognition of social equality | Whole-life value |
| Yuan & Gasco-Hernandez (2021) | “The organizers of civic hackathons were the founders of the non-profits and the CIOs (or similar) of the governments involved, showing the initiatives were legal and supported by public officials with authority.” (p.534-535) | Data collaboration between government and NPOs | Data collaboration factors | “These civic hackathons usually addressed (social) concerns of governments, which were often reflected in the mandates or plans of local authorities.” (p.535) Civic hackathons provide feasible solutions to the social problems concerned by the government, reducing the time and efforts of the government. | Efficiency | Performance value |
| Boudreau (2021) | “Through their reuse of open data, intermediaries help to transform them into intelligible information for citizens who often lack the technical skills and statistical knowledge to use and analyse them.” (p.857) | Data collaboration between government and NPOs | Data collaboration factors | “Nord-Ouvert, a Montreal-based NPO specialising in the development of civic applications, used it to advance some of its applications.” (p.862) This will save the government’s investment of money and efforts. | Efficiency | Performance value |
| De Crescenzo et al. (2021) | “Backers involved in civic crowdfunding campaigns generally expect non-financial or emotional rewards.” (p.582) | Reputational incentives | Incentivizing factors | “They are driven by a sense of commitment and belonging to local territories.” (p.582) | Self-efficacy | Whole-life value |
|  |  |  |  | “Civic crowdfunding is a new way of interacting between citizens, the public sector and crowdfunding web platforms.” (p.581) | Engagement | Administrative value |
| Mærøe et al. (2021) | “All ideas are presented in the VOLIS system for citizens to add comments, while citizen can vote for their favorite.” (p.138) | Score, comment, like and vote citizen messages | Citizen-citizen interaction factors | “Then the two ideas with the most votes are implemented.” (p.138) This indicates that citizens’ opinions have the opportunity to be adopted by the government, which makes citizens feel empowered. | Self-efficacy | Whole-life value |
| Cinderby et al. (2021) | “A design competition for University student teams to develop infrastructure and road layout solutions targeting safety and sustainability.” (p.1025) | Online competition events | Online marketing factors | “Generating new knowledge and engaging marginalised voices.” (p.1023) | Innovative solution | Performance value |
|  |  |  |  | “Engagement of University students to develop plan for road improvements responding to the co-created design brief.” (p.1025) | Engagement | Administrative value |
| Wang et al. (2021) | “With LBS, public advices can be correspondingly connected to urban spaces, thus providing more operational suggestions for the government.” (p.4) | GIS | Ease-of-use factors | With the help of GIS, users can simplify the steps of using the APP. | Good participation experience | Experiential value |
| Legard & Goldfrank (2021) | “In the Sistema, voters could simply go to the nearest ballot station (offline vote) or conveniently vote online from home or work, or wherever they happened to be with a smartphone or laptop (online vote).” (p.171) | Mix use of online/offline channels | Online/offline integration factors | “Sistema explicitly combined representative and participatory democracy.” (p.169) | Engagement  Legitimacy | Administrative value |
| Yang et al. (2021) | Use of @ function. | Dialogic loop | Government-citizen communication factors | Citizens’ ability to cope with crisis situations. | Crisis response capacity | Capacity value |
|  |  |  |  | “The correlation model showed posts related to the COVID-19 crisis that contain ‘@other accounts’ positively improve citizens’ reposts and likes, which indicated more interactions between different accounts.” (p.11)  “The increase of pictures brought a significant increase in comments.” (p.11) | Accountability  Engagement | Administrative value |
|  | Use of picture, links. | Media richness |  |  |  |  |
| Wukich (2021) | Use interrogative sentences to guide citizens to interact. | Dialogic loop | Government-citizen communication factors | Citizens’ ability to cope with crisis situations. | Crisis response capacity | Capacity value |
|  |  |  |  | “By engaging residents to share ideas, this conversation starter elicited a relatively high number of audience reactions, comments, and shares.” (p.202) | Engagement | Administrative value |
| Pihlajamaa & Merisalo (2021) | “A reward of 1500 euros for each team selected to participate in the pilot phase. The chosen participants received additional compensation of 10000– 30000 euros for their work during the pilot phase.” (p.1913) | Monetary reward | Incentivizing factors | “The primary means for motivating the participants was a public procurement of innovation contract that the winner(s) would be offered after the contest.” (p.1916) | Engagement | Administrative value |
| Bertello et al. (2021) | “EUvsVirus rewarded more than 100 ideas with over 100,000 euros pledged by partnering organizations for the hackers’ hard work.” (p.181) | Monetary reward | Incentivizing factors | “Civil society, innovators, partners, and investors across Europe invested their time and energy to develop innovative solutions for coronavirus-related challenges.” (p.180) | Innovative solution | Performance value |
| Buntaine et al. (2021) | “The platform sent reporters in treated zones 2–5 additional text messages at the end of each week emphasizing that their responses had been sent to the KCCA’s Waste Management Unit and used to develop action plans.” (p.693) | Mandatory message reply | Government-citizen communication factors | “Highlighting the importance of continuously reinforcing responsiveness in each area of government action to sustain reporting.” (p.701)  Communicative responsiveness – where citizens’ messages are acknowledged and the agency’s process of turning monitoring into action is disclosed – allows citizens to attribute any changes to the public services they observe to their reporting.” (p.693) | Engagement  Legitimacy  Accountability | Administrative value |
| Lappas et al. (2021) | “Posts in which the local government official has responded/replied to a user’s comment.” (p.94) | Dialogic loop | Government-citizen communication factors | Citizens’ ability to cope with crisis situations. | Crisis response capacity | Capacity value |
|  |  |  |  | “Commenting enables two-way communication between users and local governments.” (p.91)  “The number of comments was significantly and positively related with the use of videos.” (p.97) | Accountability  Engagement | Administrative value |
|  | Use of video. | Media richness |  |  |  |  |
| Jankowski et al. (2021) | “A softGIS survey in Finland is an online multi-page questionnaire with various open-ended and closed-ended questions, combined with an interactive map that allows marking geographical features.” (p.1534) | GIS | Ease-of-use factors | With the help of GIS, users can simplify the steps of using the APP. | Good participation experience | Experiential value |
